# Exploring endophytes for *in vitro* synthesis of bioactive compounds similar to metabolites produced *in vivo* by host plants

**DOI:** 10.3934/microbiol.2021012

**Published:** 2021-05-26

**Authors:** Hemant Sharma, Arun Kumar Rai, Divakar Dahiya, Rajen Chettri, Poonam Singh Nigam

**Affiliations:** 1 Department of Botany, Sikkim University, 6^th^ Mile Tadong, Gangtok, Sikkim, India; 2 School of Human Sciences, London Metropolitan University, Holloway Road, London, UK; 3 Department of Botany, Sikkim Government Science College, Chakung, Sikkim, India; 4 Biomedical Sciences Research Institute, Ulster University, Coleraine, Northern Ireland, UK

**Keywords:** Endophytes, biomolecules, secondary-metabolites

## Abstract

Endophytes represent microorganisms residing within plant tissues without typically causing any adverse effect to the plants for considerable part of their life cycle and are primarily known for their beneficial role to their host-plant. These microorganisms can *in vitro* synthesize secondary metabolites similar to metabolites produced *in vivo* by their host plants. If microorganisms are isolated from certain plants, there is undoubtedly a strong possibility of obtaining beneficial endophytes strains producing host-specific secondary metabolites for their potential applications in sustainable agriculture, pharmaceuticals and other industrial sectors. Few products derived from endophytes are being used for cultivating resilient crops and developing non-toxic feeds for livestock. Our better understanding of the complex relationship between endophytes and their host will immensely improve the possibility to explore their unlimited functionalities. Successful production of host-secondary metabolites by endophytes at commercial scale might progressively eliminate our direct dependence on high-valued vulnerable plants, thus paving a viable way for utilizing plant resources in a sustainable way.

## 1. Importance of medicinal plants

Different types of microbial species as symbionts of a plant, living most of their lifetime within the tissues showing no symptoms, are recognized as endophytes [1]. Normally plants have always been a primary source of food and medicine since time immemorial. Medicinal plants have always remained a primary source for treating common ailments and diseases in some parts of the world lacking basic healthcare facilities. Several allopathic drugs are either transformed or derived directly from plant parts thus putting pressure on already depleting plant resources. Alternative source of some of the metabolites commonly derived from plants would eventually reduce our dependence on plant-based bio-resources. The herbal medicines derived from plants have been well documented since ancient civilizations of India, Egypt, China, Central Asia, Greece, etc. These civilizations, over several centuries, have played a considerable role in exploring and reporting beneficial properties of diverse group of plant species [2]–[4]. Medicinal plants and their derivatives remain a major source of medicine for regular ailments in developing countries as they are reasonably priced and easily accessible [5].

Last few decades have again received a considerable interest towards the search for unique metabolites from natural sources [6]. Several components of drugs are still derived directly from plant parts while few others are transformed from the molecules obtained from various plants. Even after exploring for natural compounds all these years, plants continue to hold treasure house of unknown metabolites [7]. Demand for Ayurvedic and Chinese herbal medicines are very high due to inadequate facilities for allopathic treatment and poor healthcare system in these regions [8].

About 70% of people across the globe continue to rely on herbal medicines as remedies and for treating numerous diseases [9]. There is a considerable growth in consumption of medicines derived from plants even in Western and European countries [10]. Herbal products occupy fair share in overall drug market across the globe which will continue to grow steadily [11],[12]. Medicinal plants continue to hold a significant place in various therapeutics and health care systems leading to massive demand for plant-based bio-resources [13].

## 2. Characteristics of endophytes

The microorganisms such as fungi, bacteria including actinomycetes and viruses that reside within plant tissues are known as endophytes [14]. The endophytes have been classified as true endophytes or transient endophytes depending upon their diversity, biological nature, classification and method of transmissions [15]. Endophytes were further classified by Rodriguez *et al*. into clavicipitaceous (class 1) and non-clavicipitaceous (classes 2, 3 & 4) based upon the narrow or broad range of hosts, types of tissues colonized, pattern of colonization in plants that is either extensive or limited, *in planta* bio-diversity that could be high, low or unknown, vertical or horizontal types of transmission through different generations and habitat or non-habitat adapted fitness benefits. Tolerance to drought conditions and enhancement of growth are common non-habitat adapted benefits, irrespective of origin of habitat, whereas benefits of habitat adapted are specific to the habitat with selective pressures that include salinity, pH and temperatures [16].

**Figure 1.**
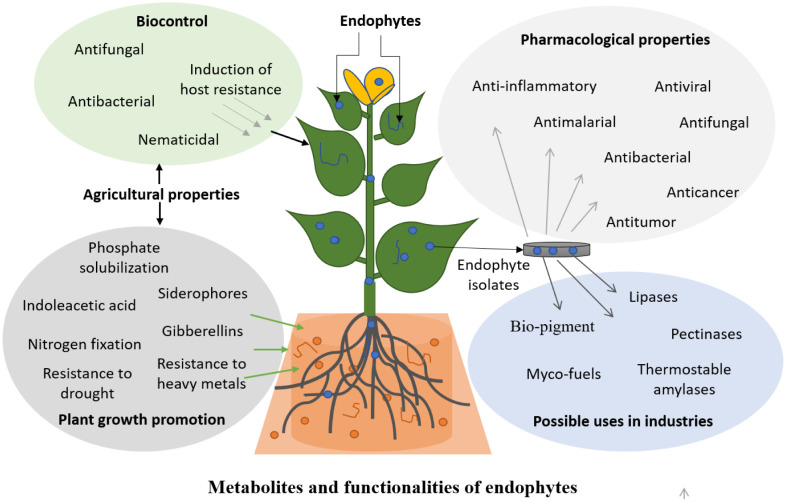
Possible applications of metabolites and functionalities derived from endophytes in different sectors.

Considerable attention in the extensive investigation of beneficial microorganisms from the plant tissues fully demonstrate their unique abilities to produce secondary metabolites of the host plant and collection of functionalities (Figure 1) with their possible applications in agriculture, pharmaceutical and industrial sectors [17]–[20]. Importance of endophytes came into light only after the demonstration of toxic syndrome in cattle caused by endophytes of pasture grasses [21],[22]. Endophytes are abundant in nature and have been found in all those plant species that have been studied so far. These microorganisms share an obligate or facultative relationship with the plant while causing no harm to their host [23]. Endophytes have characteristic of producing bioactive compounds, as they have been isolated from the tissues of roots, leaves and stems of their host plant, which produce similar metabolites [24],[25].

Identification of fungal endophytes has been carried out by studying the morphological characteristics after sporulation. However, classification of non-sporulating fungi is problematic and it is carried out through phylogenetic analyses of rDNA-ITS sequences after the amplification of DNA extracted from the fungal mycelia [26],[27]. Similarly, phylogenetic analyses of the 16s sequences obtained after the amplification of rDNA would help to identify the bacterial endophytes [28].

## 3. Metabolites and activities of endophytes

Microbial endophytes are well-known for their ability to produce a wide range of pharmacologically important compounds with enormous therapeutic potentials; which have been identified as antiviral, antifungal, antibacterial, antitumor and anticancer agents. A number of endophytes are prospective source of plant growth promoting factors, and plant hormones. They can synthesize compounds of applications in the field of agriculture, iron chelating agents, compounds with nematocidal, insecticidal activities and abiotic stress tolerant properties. Some endophytes have shown their ability to secrete wide range of extracellular enzymes, such as phosphatase enzyme to convert insoluble phosphates to soluble form for its easy assimilation by plants. Endophytes produce molecules suitable for the production of bio-fuels and degrade complex organic and inorganic substances with suitable use in industrial sectors. The useful properties of endophytes are listed below with their potential significance in respective sectors.

### 3.1. Potential significance of endophytes with respect to agriculture

Published work state that endophytes are definitely an excellent source of metabolites and desired functions that could prove to be beneficial in organic farming system. Some of the endophytes could be used as bio-pesticides against phytopathogens due to their antimicrobial, nematocidal and insecticidal qualities.

#### 3.1.1. Pesticidal properties of endophytes

The extracts from a perennial grass native to most of Europe *Phleum pratense*, demonstrated myco-toxic properties, which were secreted by a systemic grass symbiont fungal endophyte *Epichloe typhina*. The antifungal properties of the extracts were detected against *Cladosporium herbarum* [29]. A strain of fungus, L1930, obtained from *Larix laricina* displayed insecticidal property against larvae of spruce budworm. Chitinase, known to degrade chitin polymers that are essential part of a fungal cell wall, was produced by bacteria, an endophyte of *Sinapis arvensis*. The bacterial endophyte was identified as *Bacillus cereus strain* [30] and was known to play a defensive role against a phytopathogen *Rhizoctonia solani* [31].

Strain of *Neotyphodium* sp. (AR601) producing large quantities of alkaloids such as loline and ovaline inoculated into a cultivar ‘Jackal’ of turf tall fescue have shown birds deterring ability [32]. Several endophytes have consistently shown to induce effective resistance in plants against common phytopathogens, by producing proteins related to pathogenesis. Fungal endophytes found from the leaves of trees typically growing in Indian states of Western Ghats, and Tamil Nadu, were able to secrete chitinase and chitosanase, which could increase defenses in host plant against phytopathogens, by initiating host defenses and increasing resistance [33],[34].

#### 3.1.2. Plant growth promotion by endophytes

Endophytes have been identified to solubilize phosphates, produce siderophores, secrete plant growth promoting factors and increase soil nutrition by degrading complex organic molecules. It was observed that ericoid plants were able to thrive in extreme conditions due to the presence of an endophyte, *Hymenoscyphus ericae*, that produced several enzymes along with phosphate solubilization properties [35]. Lu *et al* isolated *Colletotrichum* sp. B501 from the healthy stems of *Artemisia annua* L, secreted IAA and 3β-hydroxy-ergosta-5-ene, these compounds that showed properties for plant growth [36]. The production of a range of factors and plant hormones have been reported from both fungal and bacterial endophytes [37]–[41]. Some endophytes have been found to increase tolerance of plants in soils contaminated with heavy metals [39],[42],[43]. *In vitro* investigation of endophyte-plant interaction in *Echinacea purpurea* demonstrated that colonization potential of bacterial strains belonging to *Pseudomonas* and *Arthrobacter* genus were tissue specific in host plants from which they were originally obtained but did not show similar specificity in non-host plants [44]. Further, plant growth promotion (PGP) was observed in inoculated plants due to the secretion of Indoleacetic acid by endophytic bacteria. Physiology of plants were influenced by compounds secreted by endophytes and plant metabolites, in turn, they regulated the growth of endophytes. Similarly, endophytic strains of *Bacillus* sp. isolated from *Thymus vulgaris* demonstrated plant growth promoting traits in *Solanum lycopersicum* L under salt stress along with showing antagonistic activity against *Fusarium oxysporum* and reduced the antioxidant stress on plants [45]. Antagonistic properties against human pathogens were observed in cultivable bacteria obtained from different segments *viz*. roots, stem, leaf and flower of *Origanum vulgare* L [46]. *Pseudomonas* and *Bacillus* were the most represented genera of endophytes in *Lavandula dentata* that demonstrated multiple PGP traits [47]. All these aspects make these endophytes a potential source of bio-fertilizer, bio-pesticide, plant growth promoter and maintain overall growth and development of the plants.

Some of the bacterial and fungal endophytes and their potential applications in agriculture are listed in Table 1 (1.1 and 1.2).

### 3.2. Potential significance of endophytes with respect to pharmaceuticals

Products derived from natural sources are a major area of research for discovering the range of their functions, that could be used in pharmaceutical industries [66],[67]. Microorganism from different biotypes have repeatedly proven to be a constant source of secondary metabolites with novel and unique properties, which have found a major place in medical sector [68]. Since the discovery of endophytes and their ability to produce plant secondary metabolites and other bioactive compounds, several reports are available on mining of novel secondary metabolites [69],[70]. Different saponins showing antagonism were extracted from *Fusarium* sp. PN8 isolated from *Panax notogensing* [71].

#### 3.2.1. Antimicrobial properties of endophytes

Some species of endophytes are known to produce antimicrobial compounds. Phomopsichalasin (**11**) an antimicrobial agent was extracted from *Phomopsis* sp., isolate no. MF6031 obtained from the twigs of *Salix gracilostyla* var. melanostachys. The compound **11** exhibited antibacterial activity against *Bacillus subtilis*, *Salmonella gallinarium* and *Staphylococcus aureus* with some amount of antagonism towards *Candida tropicalis* [72]. Findlay *et al* isolated an endophytic fungus from the needles of *Larix laricina* (Du Roi) K. Koch [53]. which produced 6-oxo-2-propenyl-3, 6-dihydro-2H-pyran-3-yl ester (**12**) showing antibacterial activity against *Vibrio salmonicida, S. aureus and Pseudomonas aeruginosa*.

In another study, a *Colletotrichum* sp. isolated from internal stem tissues of *Artemisia annua* L. showed antifungal, antibacterial and fungistatic properties. Its metabolites 6-isoprenylindole-3-carboxylic acid (**19**), 3β,5α-dihydroxy-6β-phenylacetyloxy-ergosta-7,22-diene (**21**), 3β-hydroxy-ergosta-5-ene (**15**), 3-oxo-ergosta-4,6,8(14),22-tetraene (**16**) and 3β,5α-dihydroxy-6β-acetoxy-ergosta-7,22-diene (**20**) showed antibacterial activity against Gram-positive and Gram-negative bacteria. Compounds **15**, **20** and **21** had antifungal properties, compound **15, 19** and **20** demonstrated fungistatic property [36]. Several other researchers have also studied endophytes possessing antimicrobial properties [71],[73]–[77].

**Table 1.1:** Bacterial endophytes with potential significance in agriculture sector.

Sl. No.	Functionalities	Endophytes	Properties	Host plant	Ref
1.	Chitinase	*Bacillus cereus* strain 65	Antifungal	*Sinapis arvensis* L.	[31]
2.	Jasmonates, Abscisic acid and phosphate solubilization	*Bacillus* sp., *Achromobacter* sp., *Alcaligenes* sp.	Plant growth and development	*Helianthus annuus* L.	[38]
3.	Leu-surfactin (**8**)	*Bacillus mojavensis* RRC 101	Biocontrol of *Fusarium verticillioides*	*Bacopa monnieri* L.	[48]
4.	Nitrogen fixation	*Rhizobium leguminosarum*	Biofertilization, increase rice yield.	*Oryza sativa* L.	[40]
5.	Phosphatases, Siderophore, Nitrogen fixation	*Rahnella* sp. and *Pseudomonas* sp.	Bio-fertilization	*Musa* L.	[49]
6.	Siderophore	*Streptomyces* sp. GMKU 3100	Promote plant growth	*Oryza sativa* L	[50]
7.	Plant growth promoting factors	*Enterobacter* sp. FD17	Enhancement of maize yield	*Zea mays* L.	[51]
8.	IAA, Siderophore, Phosphate solubilization	*Serratia* sp., *Enterobacter* sp., *Acinetobacter* sp., *Pseudomonas* sp., *Stenotrophomonas* sp., *Agrobacterium* sp., *Ochrobactrum* sp., *Bacillus* sp. *and Tetrathiobacter* sp.	Plant growth promotion in *Zea mays*.	*Zingiber officinale* Roscoe	[41]

**Table 1.2.** Fungal endophytes with potential significance in agriculture sector.

Sl. No.	Functionalities	Endophytes	Properties	Host plant	Ref
Clavicipitaceous					
1.	Ethyl trans-9.10-epoxy-ll-oxoundecanoate (**1**), Ethyl 9-oxononanoate (**2**), Ethyl azelate (**3**), Hydroxydihydrobovolide (**4**).	*Epichloe typhina*	Antifungal	*Phleum pratense* L.	[52]
2.	8,1′,5′-trihydroxy-3′,4′ dihydro-1′H-[2,4′]binapthalenyl-1,4,2′-trione (**5**)	Fungus L1930 (unidentified)	Insecticide	*Larix laricina* (Du Roi) K. Koch	[53]
3.	Phosphatase, Protease, Cellulase, Hemicellulases, Pectinolytic enzymes, Ligninase	*Hymenoscyphus ericae*	Phosphate solubilization, Protein breakdown, Cell wall lysis.	Ericoid plants	[35]
4.	Indole-3-acetic acid (IAA) and 3β-hydroxy-ergosta-5-ene (**6**)	*Colletotrichum* sp. B501	Plant growth hormone	*Artemisia annua* L.	[36]
5.	Phosphate solubilization	*Penicillium* sp.	Bio-fertilization	*Triticum aestivum* L.	[54]
6.	3-Hydroxypropionic acid (**7**)	*Phomopsis phaseoli and Melanconium betulinum strains*	Nematicidal	*Broad leaved tree of tropical rainforest, Betula pendula Roth. And Betula pubescens Ehrh*.	[55]
7.	Volatile organic compounds	*Muscodor albus*	Mycofumigation	*Cinnamomum zeylanicum* Blume	[56]
8.	Protease amylase, lipase, laccase, cellulase and pectinase.	Various fungal species	Enhance resistance of grasses to multiple stresses.	*Catharanthus roseus* L. (G. Don.), *Calophyllum inophyllum* L., *Bixa orellana* L., and *Alpinia calcarata*. Roscoe	[57]
9.	Gibberellins	*Penicillium* sp. M5.A and *Aspergillus* sp. M1.5	Promote plant growth and development.	*Monochoria vaginalis* (Burm.f.) C. Presl ex Kunth	[37]
10.	Siderophore	*Phaeotheca* sp. *Fusarium sp*., *Penicillium* sp. and *Arthrinium sp*.	Antibacterial	*Pinus sylvestris* L. and Rhododendron *tomentosum* Harmaja	[58]
11.	1,8-cineole (monoterpene) (**9**)	*Hypoxylon* sp.	Antimicrobial	*Persea indica* (L.) Spreng.	[59]
12.	Chitosanase, chitinase.	*Xylariaceae* sp., *Aureobasidium pullulans*, *Colletotrichum* sp., *Lasiodiplodia theobromae, Phomopsis* sp. and *Fusarium* sp., Botrytis sp., *Trichoderma* sp., *Alternaria* sp., *Nodulisporium gregarium*, Nigrospora *oryzae*, *Drechslera* sp., *Pithomyces* sp. *Sordaria* sp. and *Pestalotiopsis* sp.	Pathogenesis related proteins, phytoalexins and proteinase inhibitors in plants. Acts against phytophagous nematodes and plant pathogenic fungi.	Leaves of different tree species of Western Ghats.	[33]
13.	Phosphate solubilization	*Penicillium* sp.	Bio-fertilization	*Camellia sinensis* (L.) Kuntze	[60]
14.	Gibberellins and Indole acetic Acid	*Penicillium* sp. LWL3 and *Phoma glomerata*LWL2	Promote plant growth	*Cucumis sativus* L.	[61]
15.	Plant growth promoting factors	*Phoma* sp.	Bio-fertilizatzion	*Tinospora cordifolia* (Thunb.) Miers and *Calotropis procera* (Aiton) W.T. Aiton	[62]
16.	Trichodemin	*Trichoderma brevicompactum*	Antifungal against phytopathogens	*Allium sativum* L.	[63]
17.	Indole acetic acid, Gibberellins and Reactive oxygen species.	*Galactomyces geotrichum* WLL1	Promote growth of plants in heavy metal contaminated soil.	*Trapa japonica* Flerov	[42]
18.	Not identified (ethyl acetate extract)	*Aspergillus* sp. and *Emericella* sp.	Insecticidal properties	*Rhizophora mucronata* Lam.	[64]
19.	Plant Growth promotion and Resistance to heavy metals	*Phialocephala fortinii*, *Rhizodermea veluwensis*, and *Rhizoscyphus* sp.	Growth enhancement, Nutrient uptake, Decrease Heavy metal concentration	*Clethra barbinervis* Sieb. Et Zucc.	[43]
20.	Not identified (ethyl acetate extract)	Several fungal isolates belonging to Ascomycota and few Zygomycota.	Antifungal properties against root rot pathogens.	*Panax notoginseng* (Burkill) F. H. Chen ex C. Y. Wu & K. M. Feng	[34]****
Non clavicipitaceous					
21.	Indole Acetic Acid (IAA)	*Rhodotorula* sp. and *Rhodosporidium* sp.	Plant growth	*Populus* L.	[65]
22.	Plant growth promoting factors and reduce cadmium toxicity	*Piriformospora indica*	Enhance plant growth in cadmium toxic soil.	*Triticum aestivum* L.	[39]

#### 3.2.2. Other medicinal properties of endophytes

Few endophytes show the medicinal properties as anticancer and antitumor in their metabolites. An endophyte isolated from *Taxus brevifolia* Nutt., *Taxomyces andreanae,* was able to produce Taxol, the host secondary metabolite, in a broth culture medium [78]. Similarly, different metabolites with anticancer properties were obtained from the microbial species isolated from different plant species [79],[80]. A metabolite, Hypericin, with anti-viral, antimicrobial and anti-inflammatory properties was produced from a microbial strain isolated from *Hypericum perforatum* L. [81]. Lovastatin was produced in significant amount by an endophyte *Phomopsis vexans* isolated from *Solanum virginianum* L. [82].

The secondary metabolites and other functions from endophytes could have potential applications in therapeutics without causing damage to the respective plant species. The bacterial and fungal endophytes suitable for therapeutic purposes are listed in Table 2 (2.1 and 2.2).

**Table 2.1.** Bacterial endophytes with potential significance in therapeutic sector.

Sl. No.	Functionalities/ Metabolites/Compounds	Endophytes	Properties	Host plant	Ref
1.	Xiamycin (**62**), methyl ester of Xiamycin (**63**)	*Streptomyce* sp. GT2002/1503	Antiviral	*Bruguiera gymnorrhiza* (L.) Savigny	[83]
2	Agarwood	*Bacillus pumilus*.	Antimicrobial, Laxative, sedative, digestive, etc.	*Aquilaria* species	[84]

**Table 2.2.** Fungal endophytes with potential significance in therapeutic sector.

Sl. No.	Functionalities/Metabolites/Compounds	Endophytes	Properties	Host plant	Ref
Clavicipitaceous					
1.	Taxol (**10**)	*Taxomyces andreanae*	Antitumor	*Taxus brevifolia* Nutt.	[78]
2.	Phomopsichalasin (**11**)	*Phomopsis* sp. isolate no. MF6031	Antimicrobial	*Salix gracilostyla* var. melanostachys	[72]
3.	Cryptocandin (**13**)	*Cryptosporiopsis quercina*	Antimycotic	*Tvipterigeum wilfordii* Hook. f.	[85]
4.	3β,5α,6β-trihydroxyergosta-7,22-diene (**14**), 3β-hydroxy-ergosta-5-ene (**15**), 3-oxo-ergosta-4,6,8(14),22-tetraene (**16**), 3β-hydroxy-5α,8α-epidioxy-ergosta-6,22-diene (**17**), 3β-hydroxy-5α,8α-epidioxy-ergosta-6,9(11),22-triene, 3-oxo-ergosta-4-ene (**18**), 6-isoprenylindole-3-carboxylic acid (**19**), 3β,5α-dihydroxy-6β-acetoxy-ergosta-7,22-diene (**20**) and 3β,5α-dihydroxy-6β-phenylacetyloxy-ergosta-7,22-diene (**21**).	*Colletotrichum* sp.	Antibacterial, antifungal and fungistatic.	*Artemisia annua* L.	[36]
5.	7-butyl-6,8-dihydroxy-3(R)-pent-11-enylisochroman-1-one (**22**), 7-but-15-enyl-6,8-dihydroxy-3(R)-pent-11-enylisochroman-1-one (**23**), 7-butyl-6,8-dihydroxy-3(R)-pentylisochroman-1-one (**24**)	*Geotrichum* sp. Ccre7	Antifungal, antituberculous and antimalarial	*Crassocephalum crepidioides* (Benth.) S. Moore	[74]
6.	Asperfumoid (**25**), Asperfumin (**26**), Monomethylsulochrin (**27**), Fumigaclavine C (**28**), Fumitremorgin C (**29**), Physcion (**30**), Helvolic acid (**31**), 5α,8α-epidioxy-ergosta-6,22-diene-3β-ol (**32**), Ergosta-4,22-diene-3β-ol (**33**), Ergosterol (**34**), C*yclo* (Ala-Leu) (**35**) and C*yclo* (Ala-Ile) (**36**).	*Aspergillus fumigates* CY018	Antimycotic	*Cynodon dactylon* (L.) Pers.	[73]
7.	Brefeldin A (**37**)	*Cladosporium* sp.	Antimicrobial	*Quercus variabilis* Blume	[75]
8.	Ampelopyrone (**38**), macrosporin (**39**), 3-O-methylalaternin (**40**), methyltriacetic lactone (**41**), citreoisocoumarin, macrosporin (**42**), 3-O-methylalaternin (**43**), desmethyldiaportino (**44**), desmethyldichlorodiaportin (**45**), ampelanol (**46**), altersolanol A (**47**), alterporriols D (**48**), alterporriols E (**49**) and altersolanol J (**50**).	*Ampelomyces* sp.	Cytotoxic and antimicrobial	*Urospermum picroides* (L.) Scop. ex F.W. Schmidt	[86]
9.	Paclitaxel (**51**)	*Fusarium solani*	Anticancer	*Taxus celebica* (Warb.) H. L. Li	[79]
10.	Usnic acid (**52**), Cercosporamide (**53**), Phomodione (**54**).	*Phoma* sp. isolate No. 2323	Antibacterial	*Saurauia scaberrinae* Hemsley	[77]
11.	Phomopsin A (**55**), Phomopsin B (**56**), Phomopsin C (**57**), Cytosporone B (**58**), Cytosporone C (**59**)	*Phomopsis* sp. ZSU-H76	Antifungal	*Excoecaria agallocha* L.	[87]
12.	Not identified	*Fusarium* sp. DF2	Antimicrobial	*Taxus wallichiana* Zucc.	[88]
13.	Deoxypodophyllotoxin (**61**)	*Aspergillus fumigatus* Fresenius	Anticancer	*Juniperus communis* L. Horstmann	[80]
14.	Benquinol (**64**), Benquoine (**65**)	*Phomopsis* sp. CMU-LMA	Antibacterial and cytotoxic	*Alpinia malaccensis* (Burm. f.) Roscoe	[89]
15.	Terpene (**66**)	*Phomopsis* sp.	Antibacterial	*Allamanda cathartica* L.	[90]
16.	8-octadecanone (**67**), 1-tetradecene (**68**), 8-pentadecanone (**69**), octylcyclohexane (**70**) and 10-nonadecanone (**71**).	*Fusarium solani*	Antimicrobial	*Taxus baccata* L.	[91]
17.	Emerimidine A (**72**), Emerimidine B (**73**), Emeriphenolicins A (**74**), Emeriphenolicins D (**75**), Aspernidine A (**76**), Aspernidine B (**77**), Austin (**78**), Austinol (**79**), Dehydroaustin (**80**), and Acetoxydehydroaustin (**81**)	*Emericella* sp. (HK-ZJ)	Antiviral	*Aegiceras corniculatum* (L.) Blanco	[92]
18.	Guignardin A (**82**), Guignardin B (**83**), Guignardin C (**84**), Guignardin D (**85**), Guignardin E (**86**), Guignardin F (**87**), Palmarumycin C1 (**88**), BG1 (**89**) and JC1(**90**).	*Guignardia* sp. KcF8	Antimicrobial, Cytotoxic, Protein inhibitor	*Kandelia candel* (L.) Druce	[93]
19.	Lovastatin (**91**)	*Phomopsis vexans*	Lower blood cholesterol	*Solanum xanthocarpum*	[82]
20.	Unknown	*Luteibacter* sp. NORREL-Li2	Bio convert major ginsenosides into minor ginsenoside	*Platycodong randiflorum* (Jacq.) A. DC.	[94]
21	Saponins	*Fusarium* sp. PN8 and *Aspergillus* sp. PN17	Antimicrobial	*Panax notoginseng* (Burkill) F. H. Chen ex C. Y. Wu & K. M. Feng	[71]
Not identified					
22.	Protocatechuic acid (**92**) and acropyrone (**93)**.	Fungal endophyte	Antibacterial	*Citrus jambhiri* Lush.	[76]
23.	Hypericin (**60**)	INFU/Hp/KF/34B	Antibiotic, antiviral, anti-inflammatory, seasonal effective disorder, relief from sinusitis	*Hypericum perforatum* L.	[81]
24.	6-oxo-2-propenyl-3,6-dihydro-2H-pyran-3-yl ester (**12**)	L1930 (unidentified)	Antimicrobial	*Larix laricina* (Du Roi) K. Koch	[53]

**Table 3.1.** Bacterial endophytes with potential significance in industrial sectors.

Sl. No.	Functionalities	Endophyte	Properties	Host plant	Ref
1.	Pectinase	*Paenibacillus amylolyticus*	Pectin lyase	*Coffea Arabica* L.	[95]
2.	Thermostable α-amylase	*Nocardiopsis* sp.	Starch degradation	*Pachyrhizus erosus* L.	[96]
3.	Thermostable glucoamylase	*Streptosporangium* sp.	Starch degradation	*Zea mays L*.	[97]
4.	Protease	*Bacillus halotolerans* strain CT2	Alkaline protease	*Solanum tuberosum* L.	[98]

**Table 3.2:** Fungal endophytes with potential significance in industrial sectors.

Sl. No.	Functionalities	Endophyte	Properties	Host plant	Ref
Clavicipitaceous					
1.	Amylase, cellulase, xylanase and ligninase.	*Fusarium* sp., *Phomopsis* sp. *Phoma* sp., *Colletotrichum* sp.,	Wood degradation	*Brucea javanica* (L.) Merr.	[99]
2.	Microbial oil and cellulase	*Phomopsis, Cephalosporium, Microsphaeropsis, and Nigrospora*.	Production of bio-fuel	*Taxus chinensis* var. mairei Mast, *Cupressus torulosa* D. Don, *Keteleeria davidiana* varchienpeii, *Sabina chinensis* cv. Kaizuca and *Keteleeria evelyniana* Mast.	[100]
3.	Myco-diesel	*Gliocladium roseum* (NRRL 50072)	Energy production and utilization	*Eucryphia cordifolia* Cav.	[101]
4.	1,4-Cyclohexadi-ene (**94**)	*Hypoxylon* sp.	Oxidizes to benzene (component of crude oil)	*Persea indica* (L.) Spreng.	[59]
5.	Polyurethanases	*Pestalotiopsis microspora* E2712A	Degrade polyester polyurethane	Ecuadorian Amazonian plant	[102]
6.	Lipase	*Candida guillermondi*	Synthesis of methyl oleate	*Ricinus communis* L.	[103]
7.	Amylase	*Alternaria* sp., Phoma sp., *Nigrospora* sp.	Starch hydrolysis at alkaline pH and low temperature	*Eremophilia longifolia* (R.Br.) F. Muell.	[104]
8.	Bio-pigment	*Phoma* sp.	Bio-pigment production	*Clerodendrum viscosum* L.	[105]
9.	Xylanases	*Trichoderma harzianum*	Xylan degrading enzyme	*Sargassum wightii*	[106]
10.	Laccase	*Hormonema* sp. and *Pringsheimia smilacis*	Degrade Lignin	*Eucalyptus globules* Labill.	[107]
11.	Cellulase and Xylanase	*Acremonium* sp. *Aspergillus* sp.	Degrade cellulose and Xylan	*Memecylon excelsum* Blume, *Glochidion borneese* Mull. Arg.) Boerl.	[108]

Non clavicipitaceous					
12.	Lignocellulolytic enzymes	*Bjerkandera sp*.	Wood degradation	*Drimys winteri* J. R. Forst. & G. Forst. and *Prumnopitys andina* (Poepp. ex Endl.) de Laub.	[109]
13.	Microbial oil and cellulase	*Sclerocystis*	Production of bio-fuel	*Taxus chinensis* var. mairei Mast, *Cupressus torulosa* D. Don, *Keteleeria davidiana* varchienpeii, *Sabina chinensis* cv. Kaizuca and *Keteleeria evelyniana* Mast.	[100]

### 3.3. Potential significance of endophytes in industries

Microorganisms and their derivatives play a significant role in processing of substrate into several products for use in industrial sectors. There are many reports of enzymes being produced by endophytes isolated from different plant species. Enzymes like amylase, pectinase and lipases obtained from different endophytes have been known to hydrolyze starch, pectin and oils, respectively. Other enzymes include cellulases, xylanases, amylase, laccase, and proteases, which have application in various industrial sectors [98],[104],[106],[107]. An endophyte, *Nocardiopsis* sp., isolated from *Pachyrhizus erosus* L., was found to secrete a thermostable α-amylase, which is useful for starch degradation [96]. Similarly, *Candida guillermondii* from *Ricinus communis* L. produce lipase and helps in the synthesis of methyl oleate [103].

Some endophytes are also known to produce bio-fuels, as alternate source of conventional fuels. A fungal isolate, *Hypoxylon* sp. from *Persea indica* (L.) Spreng. was found to secrete 1,4-Cyclohexadi-ene (**94**). The compound **94** readily oxidizes to benzene, which is a main component of crude oil [59]. In another work, an endophyte *Gliocladium roseum* (NRRL 50072) isolated from *Eucryphia cordifolia* Cav. produced a bio-fuel known as myco-diesel [101].

While some of the endophytes are known to degrade polyurethane which are of great value to the industrial sector [102], others are known to produce pigment suitable for use in food industry [105]. Bacterial and fungal endophytes with their ability to produce bioactive compounds with their potential applications in industries are listed in Table 3 (3.1 and 3.2).

### 3.4. Understating the potential of endophytes through genome mining

Some microorganisms are known to synthesize only a limited number of secondary metabolites (SMs) as compared to the ones estimated through genome mining [110]. SMs are synthesized through pathways that utilize multiple enzymes. Biosynthetic gene cluster (BGC) comprises set of genes that encode for proteins required during a pathway. Diverse methods could be employed to activate the cluster of genes that remain silent under *in vitro* conditions. The genome mining approach could reduce the time taken to identify the putative genes required for the synthesis of secondary metabolites [110]. The sequencing of genes have helped in the identification of genes related to SMs and enhanced the characterization process [111]. Nielsen and Nielsen have suggested three approaches for understanding the unknown BGCs that include targeted approach: where the similar BGCs are compared to form a probable BGCs, untargeted approach involves the use of different databases to mine for information and lastly through the use of metabolomics techniques [112]. Wang et al. [113] developed bacteriophage recombinases to quickly identify and stimulate BGCs that are cryptic in strains of *Burkholderia* species. Poplar trees augmented with a modified strain of endophyte, *Pseudomonas putida* W619-TCE, showed increased reduction (90%) of trichloroethylene evapotranspiration under field tests [114].

## 4. Progress and developments

In an effort to meet the increasing demand of food and feeds, chemical fertilizers and pesticides have been commonly used in agricultural system for improving soil fertility and controlling pests, respectively. The adverse effect of use of toxic chemicals in agriculture has resulted in increasing interest in sustainable farming practices [115],[116]. Biofertilizers and Biopesticides derived from microorganisms have been effective in dealing with phytopathogens as well as Biofertilization of the soil. Bacterial and fungal endophytes have shown positive effects in plant growth promotion, pest management and improving soil health [117],[118]. Numerous endophytes have shown their ability to promote plant growth and antagonism against phytopathogens under *in vitro* conditions. Some of the strains have found their place in modern agricultural practices, such as perennial ryegrass (PRG) due to its endophyte, *Neotyphodium lolii*, was able to protect the host plant from Argentine stem weevil infection without producing any toxic compounds harmful to livestock. A product of Rye grass, AR1, infected with the endophyte has been beneficial for livestock production in places with lesser number of black beetles. Another strain of PRG, *Endosafe*, has shown better survival response in places dominated with black beetles but with decreased biomass production compared to AR1 [119].

Adaptive Symbiotic Technologies in Seattle, USA, have developed several products under the brand name BioEnsure® using a combination of beneficial endophytes. The products are able to induce tolerance in crops to drought, high salt concentration and temperature; it improves water utilization by plants and is fairly stable in different climate and soil types. The microbial formulations can easily be applied to fields along with other agriculture inputs and are non-competitive against other normal microbial flora of the soil. The products have a viability of more than two years at 4 °C. BioEnsure® products not genetically modified are classified as organic products by Organic Material Review Institute, Eugene, USA [120]. *Muscodor albus* isolated from cinnamon tree, has shown properties related to bio-fumigation and it may replace the use of methyl bromide for fumigation of soils [121]. Though the effects of endophytes cannot substitute chemical inputs altogether, combination of different methods and suitable endophyte-plant combinations could be considered for integrated pest management programs [122].

## 5. Constrains in commercialization of endophytes

There have been numerous reports on the production of plant secondary metabolites by endophytes outside its host but there are no products as such that have successfully been produced in mass scale and commercialized. Production of Taxol by endophytic fungus in the early 90s was thought to develop the process of obtaining metabolites from endophytes with eventual decrease in over-use of plants. However, apart from the use of few endophytes in agricultural system, not a single product from endophytes has made it to the market with a significant advance in secondary metabolite industry [123].

The reason for the production of host metabolites by the endophytes could be hidden in their genes that must have undergone genetic recombination during the time of their evolution [124]. Our inability to understand the mechanisms by which these endophytes function inside the host, and as stated by Bailey *et al* their evolutionary significance [125] has limited our knowledge.

Some of the constraints involved in the production of secondary metabolites under laboratory conditions include: the low-yield of secondary metabolites, optimization of growth-conditions involving variety of abiotic factors and silent gene clusters, synthesis of metabolites with unidentified functions, unclear understanding of pathways involved in the production of metabolites, role of secondary metabolites in different pathways and lack of a complete knowledge on secondary metabolites [110]. The cellular relationship between the host and its endophyte limits our ability to understand the mechanism of host-secondary metabolite production by an endophyte, and the eventual reduction in synthesis when outside its host *in vitro* system.

## 6. Future perspectives

Endophytic microorganisms have convincingly demonstrated their remarkable ability to typically produce an abundance of pharmacological metabolites with possible usage in drug manufacturing. Extensive search for newer metabolites is important to deal with multi-drug resistant microorganisms and to find alternative therapeutic drugs for several diseases. Secretion of plant growth-promoting factors and antagonistic agents against phytopathogens could easily substitute chemical inputs in sustainable agriculture practices with suitable endophytes. Novel enzymes with better specific-activity obtained from endophytes could be valuable in fermentation industries. However, most of the published findings are from controlled experiments and similar results from *in vivo* trials could satisfactorily establish the practical possibility of endophytes commercialization. Specific mechanisms involved in the complex interactions, types of selection pressures that properly govern the crosstalk between endophytes and their suitable host, efficient production of host secondary metabolites, and possible ways to effectively manipulate the biochemical-pathways, would undoubtedly require comprehensive understanding before the successful commercialization of bioactive metabolites from endophytes.

## 7. Conclusions

It is estimated that there are more than quarter million species of plants in this planet with a possibility of obtaining more than one million endophytes from these plants. Very few of these microorganisms from their diverse group have been isolated and studied so far. Apart from producing array of metabolites and functions advantageous to its host plant, these microbial resources have proven to secrete similar secondary metabolites even outside its host using *in vitro* systems. These properties of endophytes not only make them suitable candidates for exploring their ability to produce various bioactive compounds, enzymes, and biopigments, etc., but it may also reduce the dependency of humans on endangered plant species for their secondary metabolites, thus resulting in sustainable use of plant-based bio-resources. The necessary factors controlling growth of endophytes for biosynthesis of host secondary metabolites *in vitro*, are required to be optimised for commercial-scale production of plant-derived natural compounds employing these endophytes.
