# Supplementary material for: Exploring endophytes for in vitro synthesis of bioactive compounds similar to metabolites produced in vivo by host plants
Source: AIMS Microbiol. 2021 May 26;7(2):175–99. doi: 10.3934/microbiol.2021012 (PMC8255908; doi:10.3934/microbiol.2021012)
Supplement: Supplementary file 1 [file microbiol-07-02-012-s001.pdf]

Microbiology

AIMS Microbiology, 7(2): 175–199.
DOI: 10.3934/microbiol.2021012
Received: 04 February 2021
Accepted: 19 May 2021
Published: 26 May 2021

http://www.aimspress.com/journal/microbiology

Mini Review

Exploring endophytes for *in vitro* synthesis of bioactive compounds similar to metabolites produced *in vivo* by host plants

Hemant Sharma[1], Arun Kumar Rai[1], Divakar Dahiya[2], Rajen Chettri[3] and Poonam Singh Nigam[4,]*

[1] Department of Botany, Sikkim University, 6th Mile Tadong, Gangtok, Sikkim, India
[2] School of Human Sciences, London Metropolitan University, Holloway Road, London, UK
[3] Department of Botany, Sikkim Government Science College, Chakung, Sikkim, India
[4] Biomedical Sciences Research Institute, Ulster University, Coleraine, Northern Ireland, UK

***Correspondence:** p.singh@ulster.ac.uk.

2 -D structures of compounds obtained from endophytes

1. Ethyl trans-9,10-epoxy-11-oxoundecanoate [1]

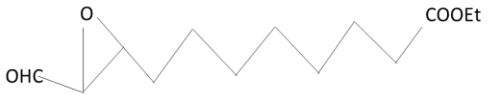

2. Ethyl 9-oxononanoate [2]

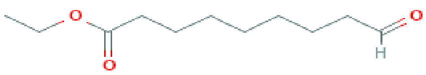

3. Ethyl zaelate [1]

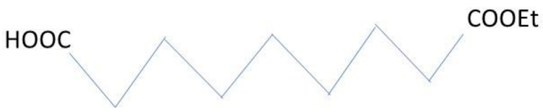

4. Hydroxydihydrobovolide [3]

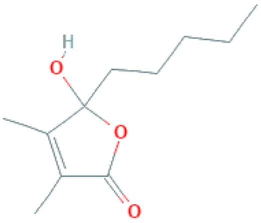

5. 8,1’,5’-trihydroxy-3’,4’ dihydro-1’H-[2,4’]binapthalenyl-1,4,2’-trione

6. 3β-hydroxy-ergosta-5-ene [4]

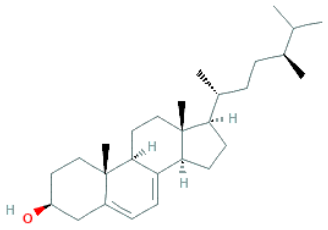

7. 3- Hydroxypropionic acid [5]

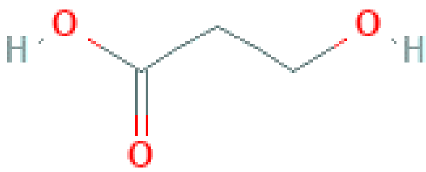

8. Leu-surfactin

9. 1,8-cineole (monoterpene) [6]

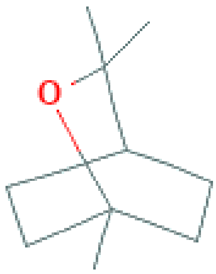

10. Taxol [7]

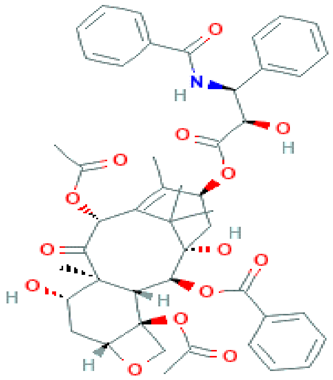

11. Phomopsichalasin [8]

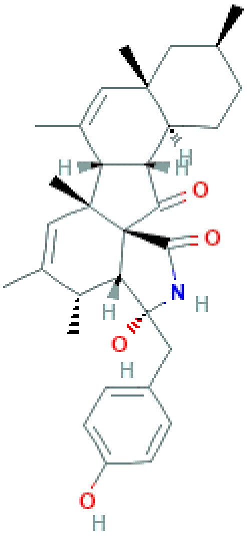

12. 6-oxo-2-propenyl-3,6-dihydro-2H-pyran-3-yl ester

13. Cryptocandin [9]

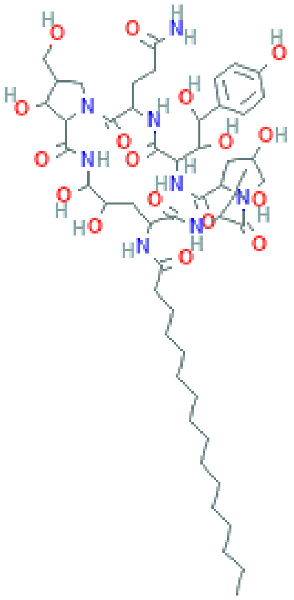

14. 3β,5α,6β-trihydroxyergosta-7,22-diene

15. 3β-hydroxy-ergosta-5-ene

16. 3-oxo-ergosta-4,6,8(14),22-tetraene

17. 3β-hydroxy-5α,8α-epidioxy-ergosta-6,22-diene

18. 3β-hydroxy-5α,8α-epidioxy-ergosta-6,9(11),22-triene, 3-oxo-ergosta-4-ene

19. 6-isoprenylindole-3-carboxylic acid [10]

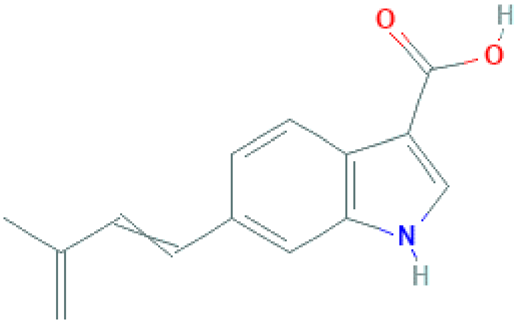

20. 3β,5α-dihydroxy-6β-acetoxy-ergosta-7,22-diene

21. 3β,5α-dihydroxy-6β-phenylacetyloxy-ergosta-7,22-diene

22. 7-butyl-6,8-dihydroxy- 3(R)-pent-11-enylisochroman-1-one [11]

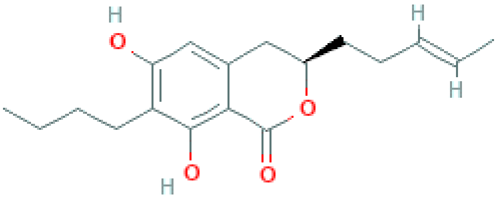

23. 7-but-15-enyl-6,8-dihydroxy-3(R)-pent-11-enylisochroman-1-one [12]

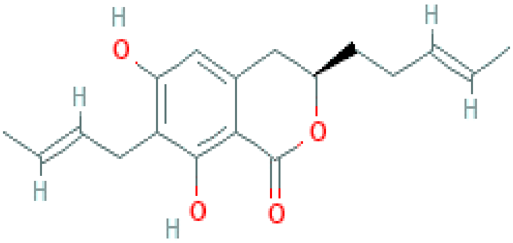

24. 7-butyl-6,8-dihydroxy-3(R)-

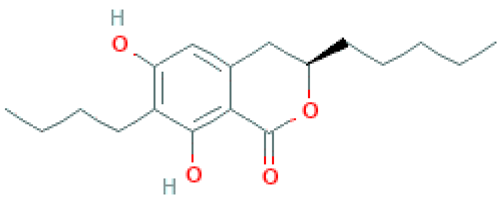

25. Asperfumoid [14]

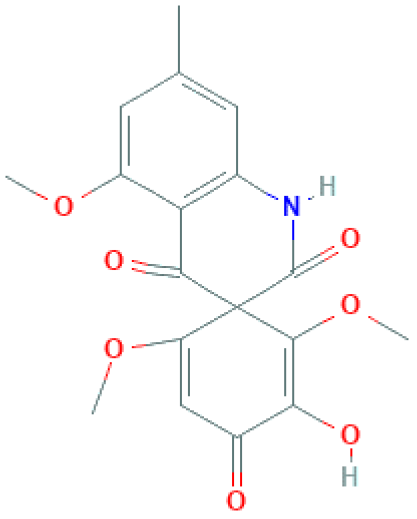

26. Asperfumin [15]

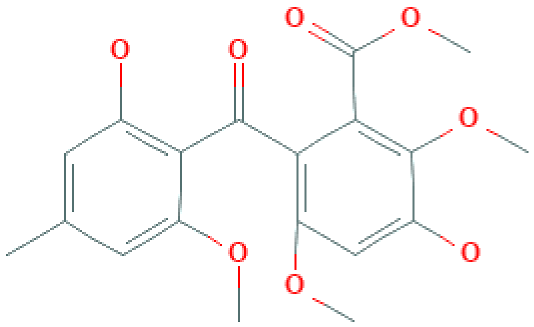

27. Monomethylsulochrin [16]

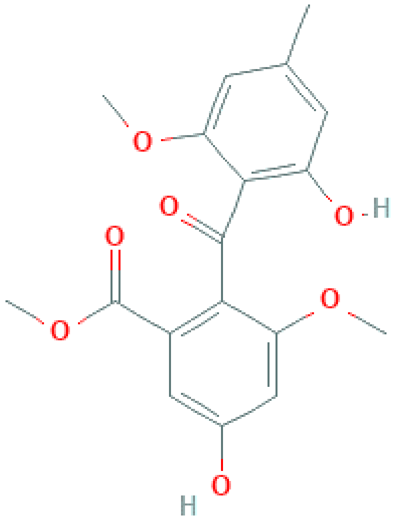

28. Fumigaclavine C [17]

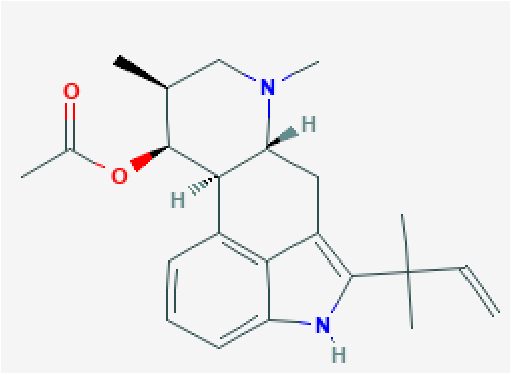

29. Fumitremorgin C [18]

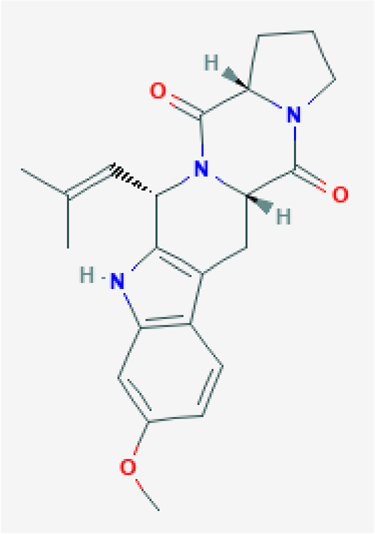

30. Physcion [19]

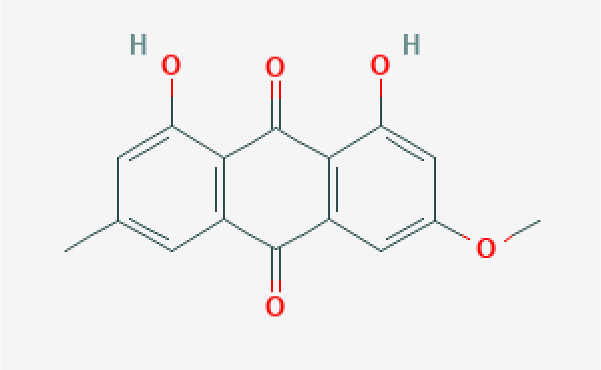

31. Helvolic acid [20]

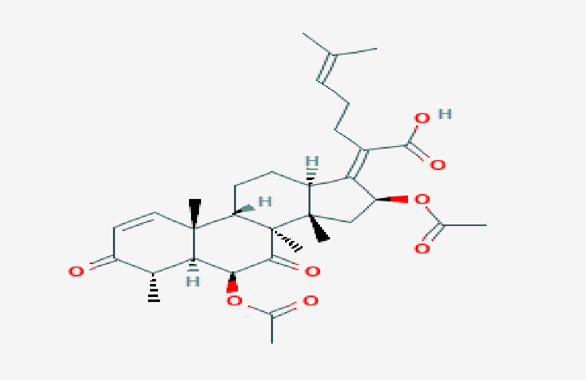

32. 5α,8α-epidioxy-ergosta-6,22-diene-3β-ol [21]

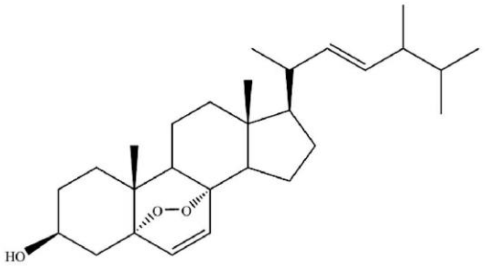

33. Ergosta-4,22-diene-3β-ol

34. Ergosterol [22]

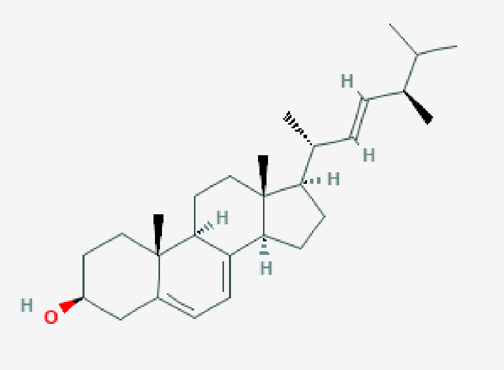

35. *Cyclo* (Ala-Leu)

36. *Cyclo* (Ala-Ile)

37. Brefeldin A [23]

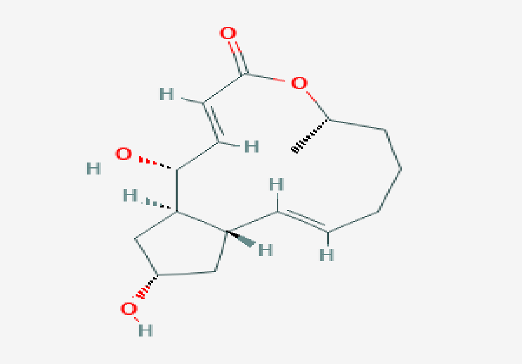

38. Ampelopyrone [24]

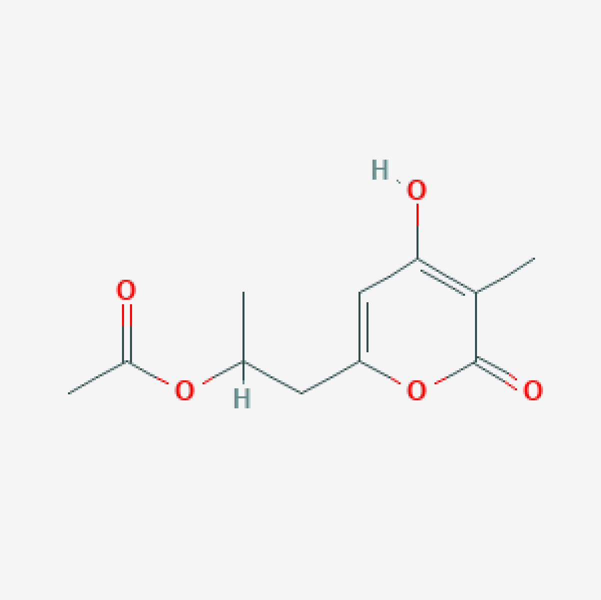

39. Macrosporin [25]

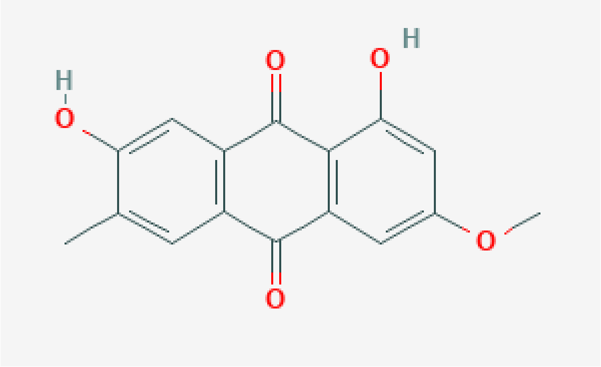

40. 3-O-methylalaternin

41. Methyltriacetic lactone

42. Citreoisocoumarin [26]

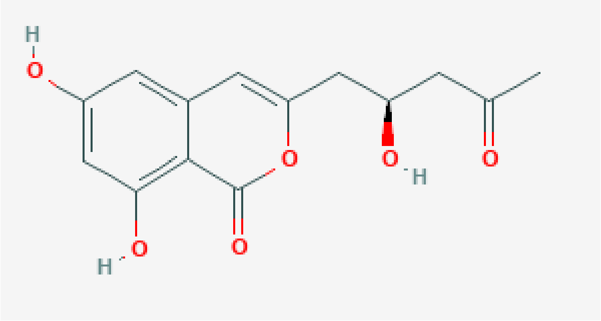

43. Macrosporin [27]

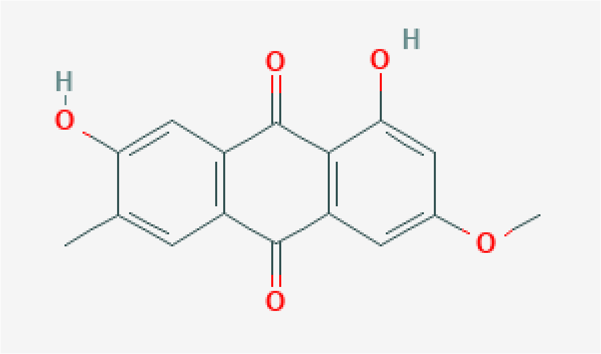

44. Desmethyldiaportino

45. Desmethyldichlorodiaportin [28]

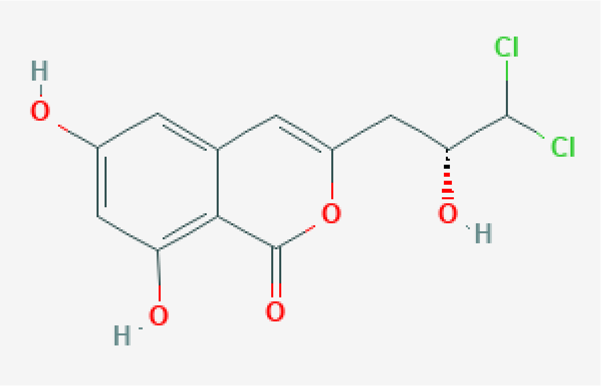

46. Ampelanol [29]

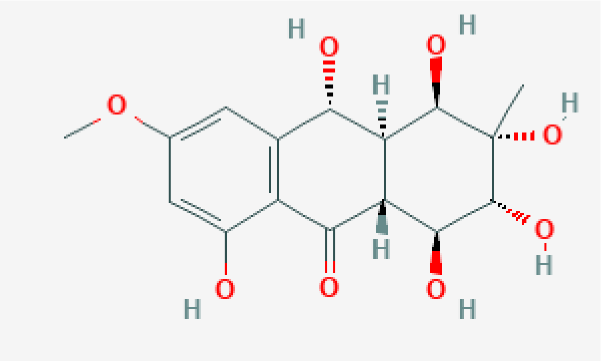

47. Altersolanol A [30]

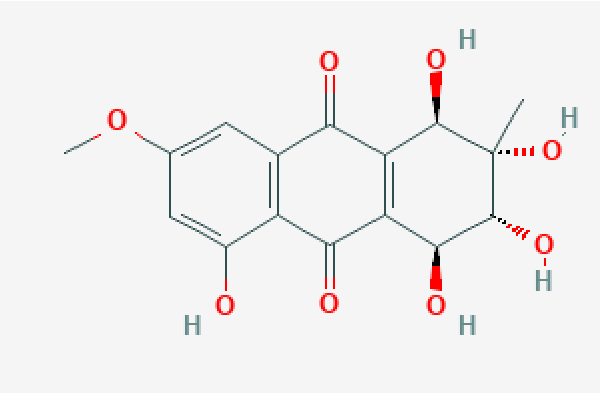

48. Alterporriols D

49. Alterporriols E [31]

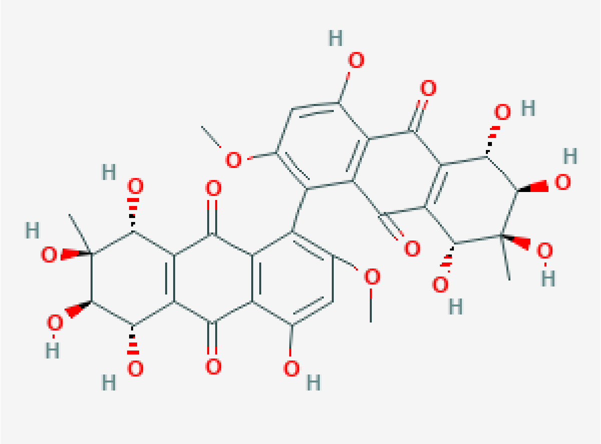

50. Altersolanol J [32]

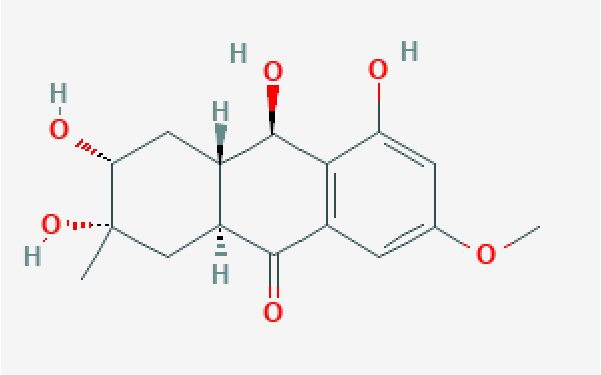

51. Paclitaxel [33]

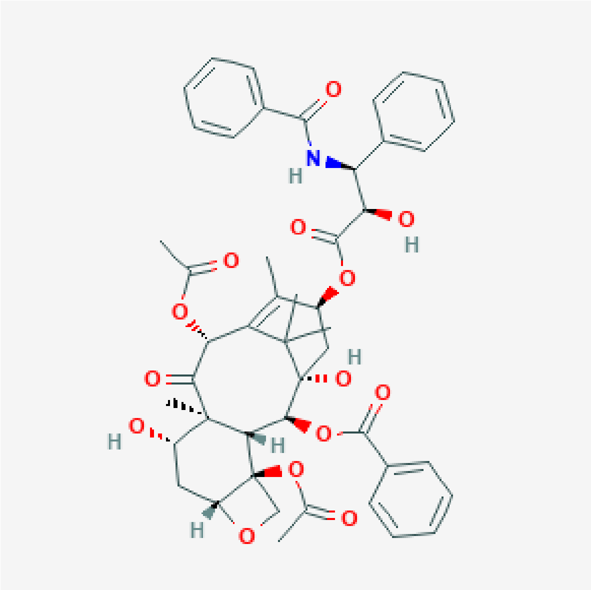

52. Usnic acid [34]

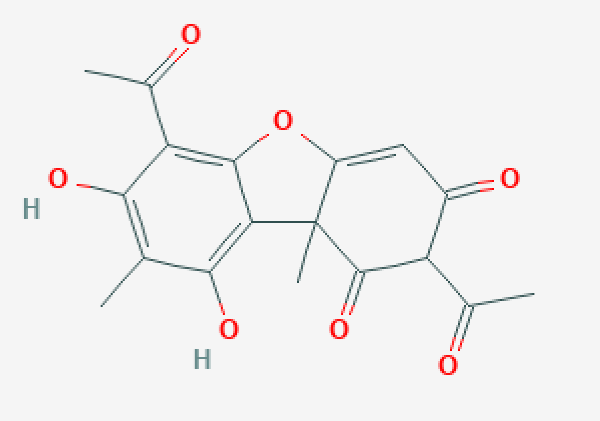

53. Cercosporamide [35]

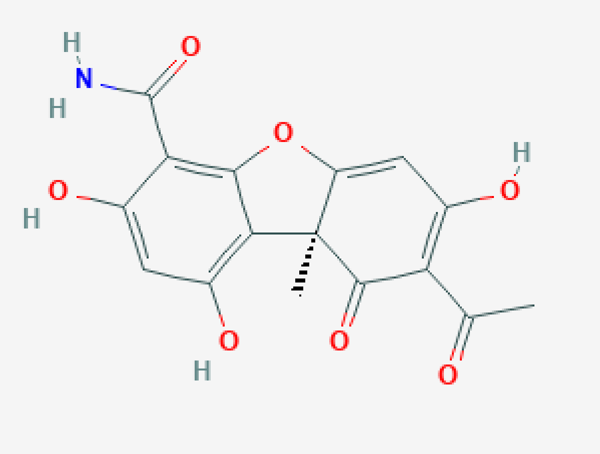

54. Phomodione [36]

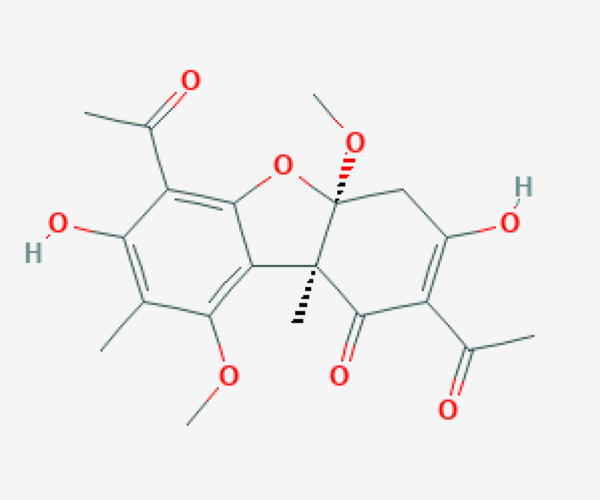

55. Phomopsin A [37]

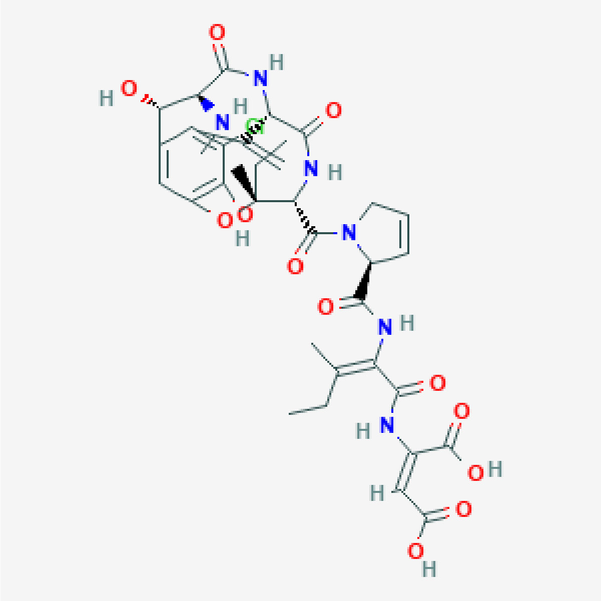

56. Phomopsin B [38]

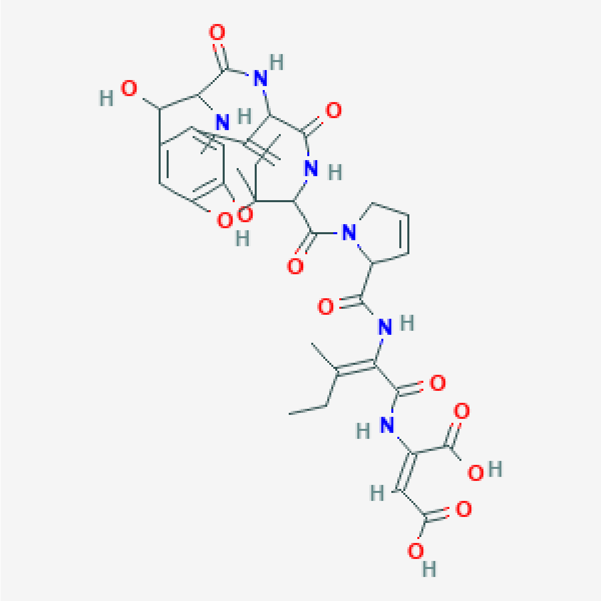

57. Phomopsin C [39]

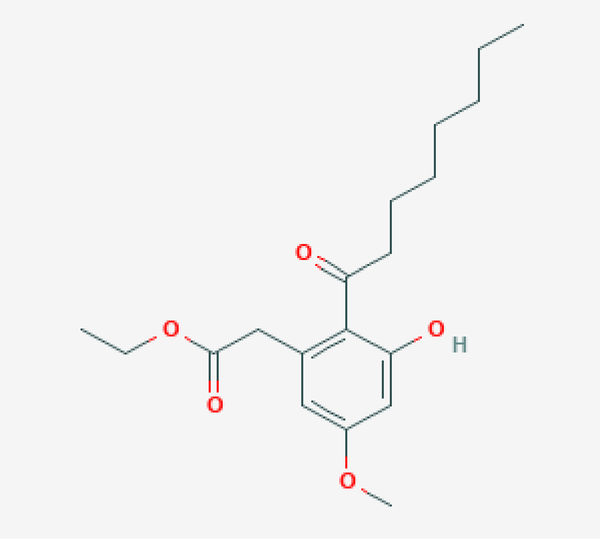

58. Cytosporone B[40]

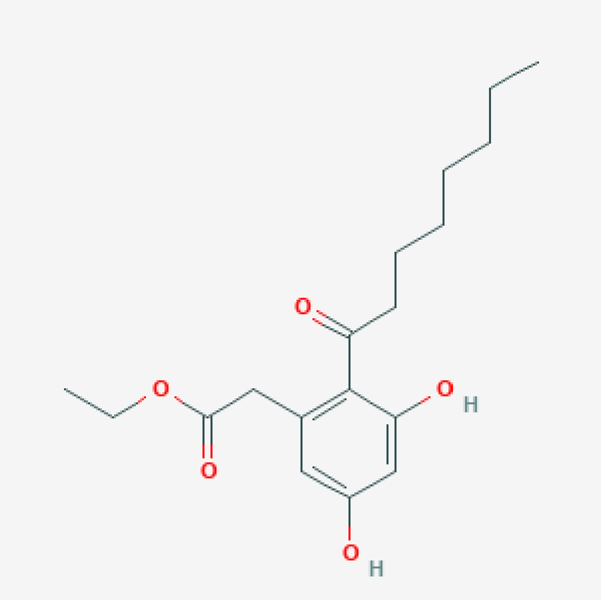

59. Cytosporone C [41]

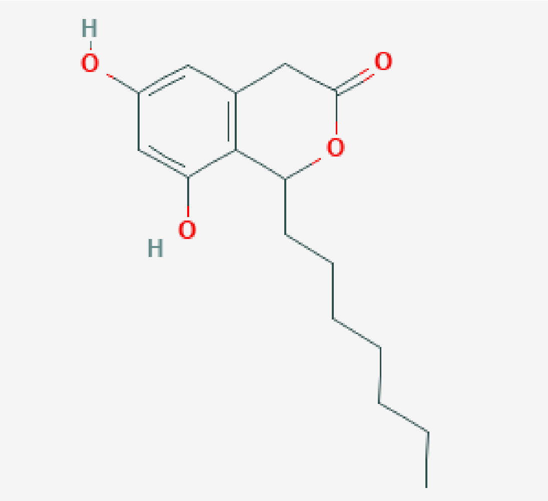

60. Hypericin [42]

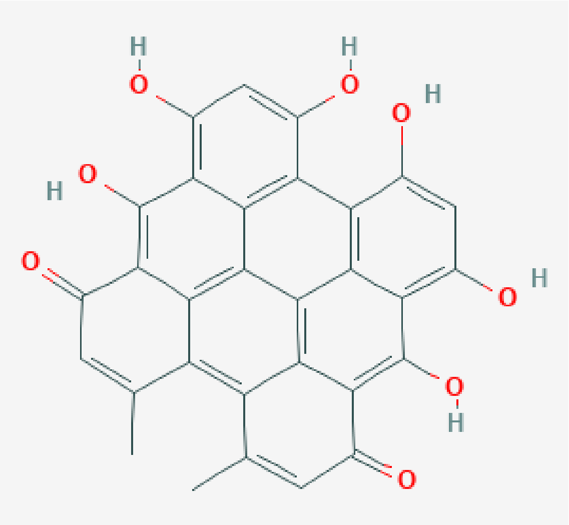

61. Deoxypodophyllotoxin [43]

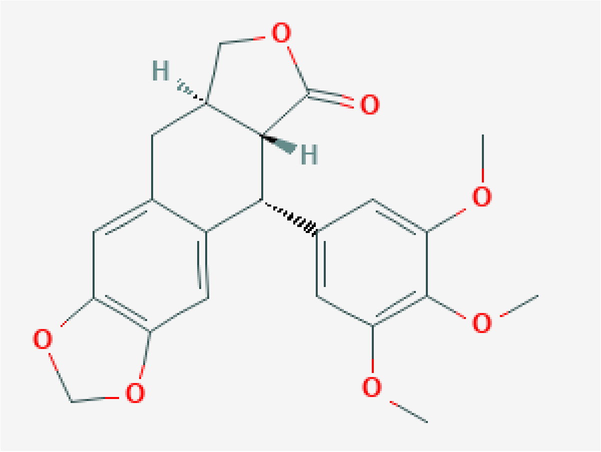

62. Xiamycin [44]

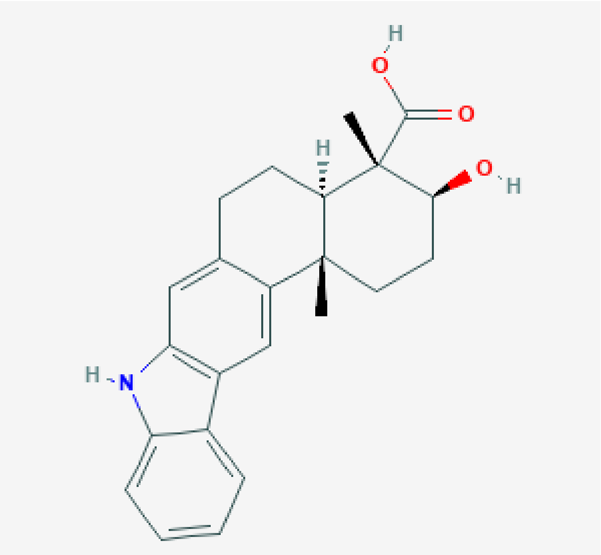

63. Methyl ester of Xiamycin [45]

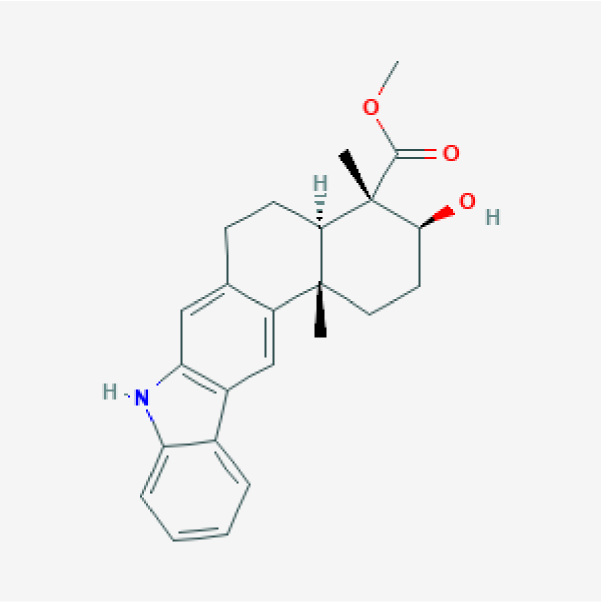

64. Benquinol [46]

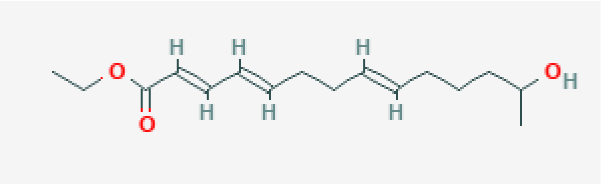

65. Benquoine [47]

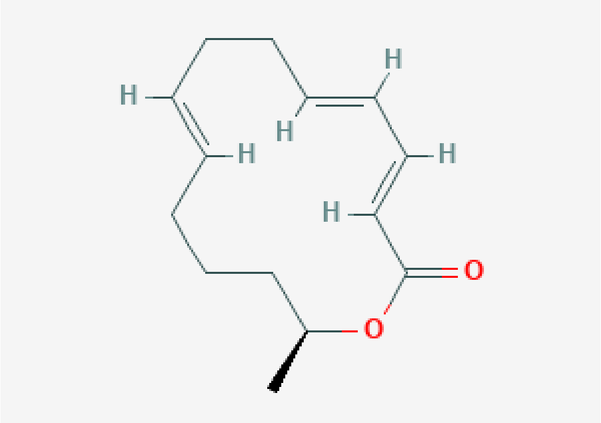

66. Terpene [48]

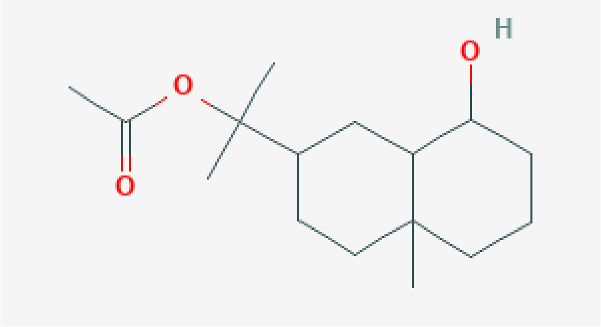

67. 8-octadecanone [49]

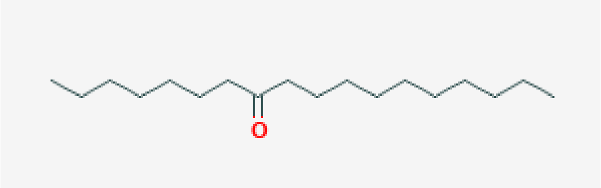

68. 1-tetradecene [50]

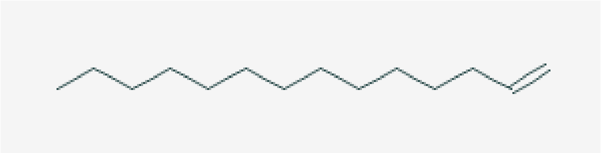

69. 8-pentadecanone [51]

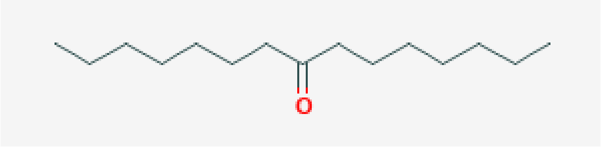

70. Octylcyclohexane [52]

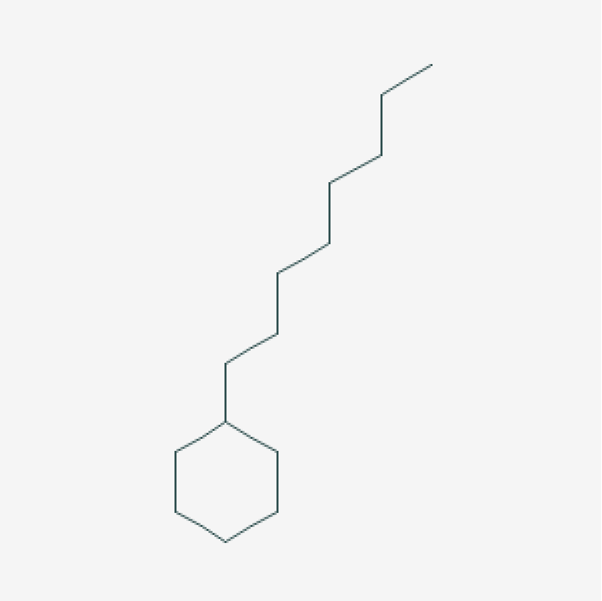

71. 10-nonadecanone [53]

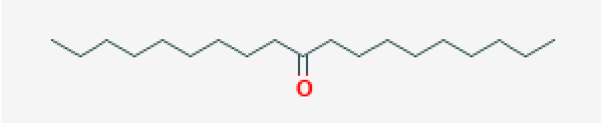

72. Emerimidine A [54]

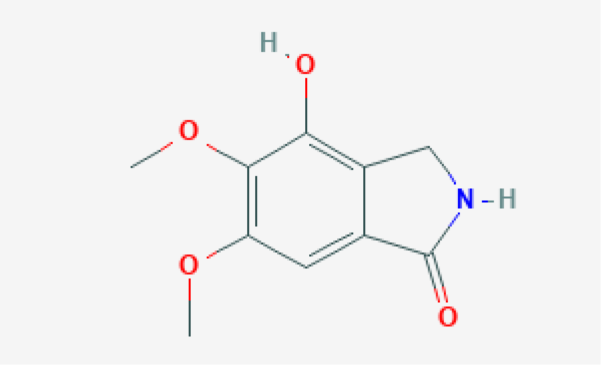

73. Emerimidine B [55]

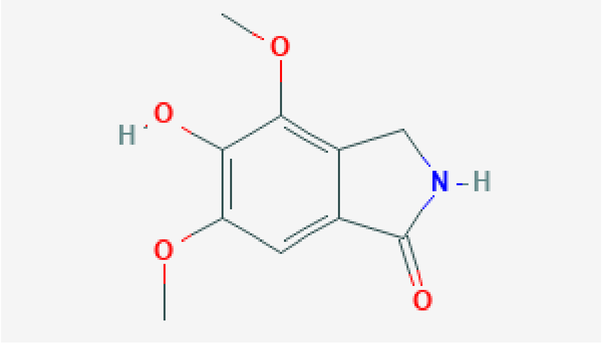

74. Emeriphenolicins A [56]

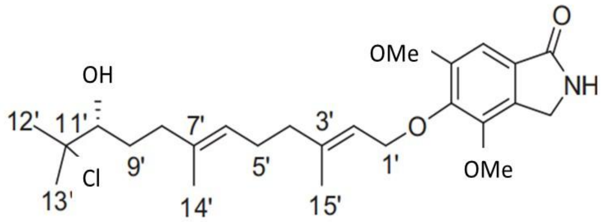

75. Emeriphenolicins A [56]

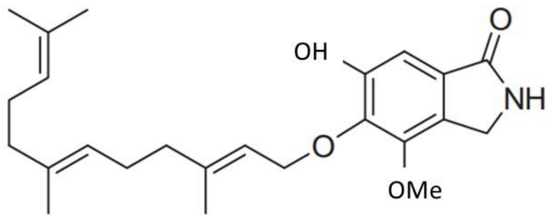

76. Aspernidine A [57]

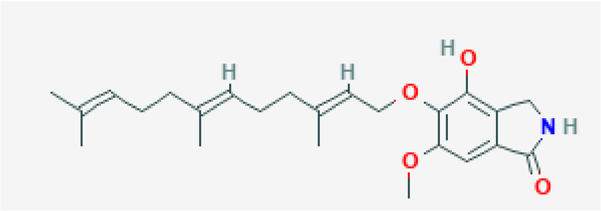

77. Aspernidine B [58]

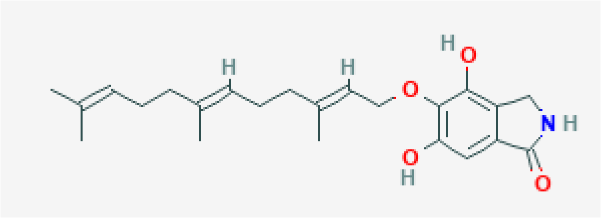

78. Austin [59]

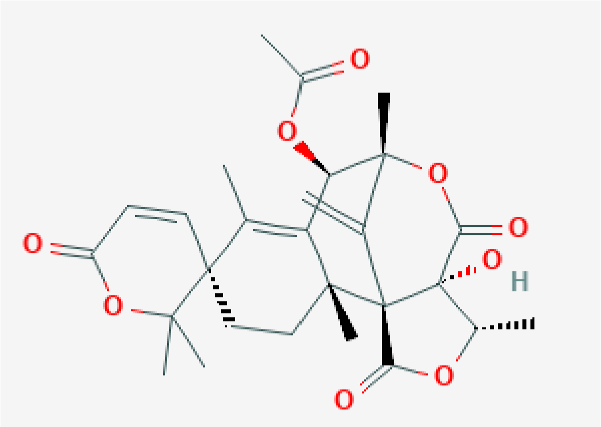

79. Austinol [60]

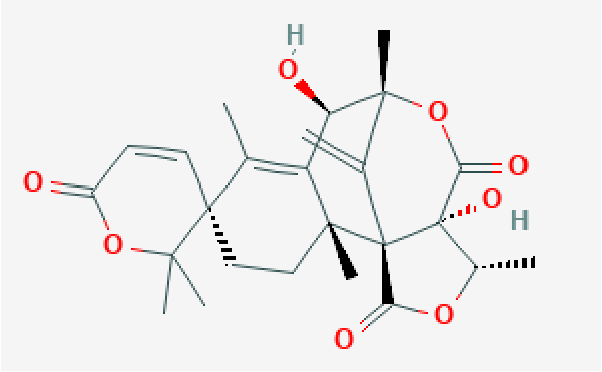

80. Dehydroaustin [61]

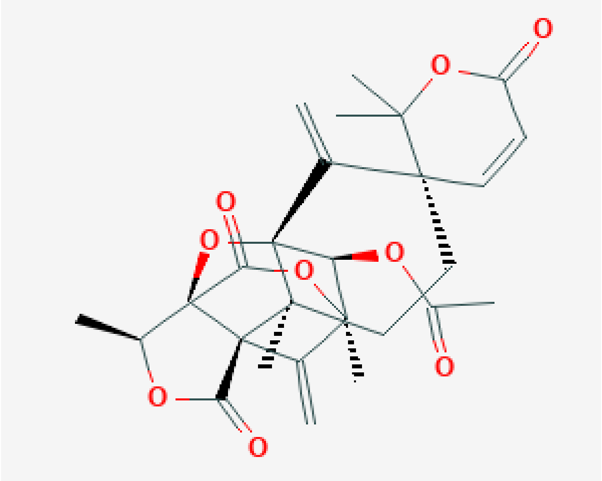

81. Acetoxydehydroaustin [62]

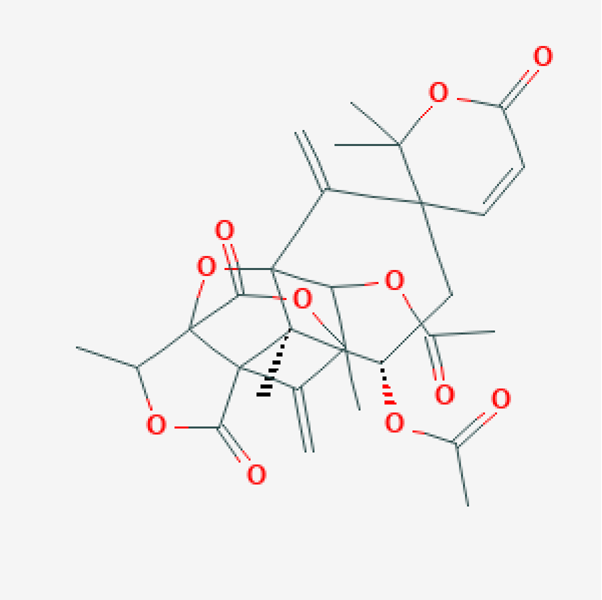

82. Guignardin A [63]

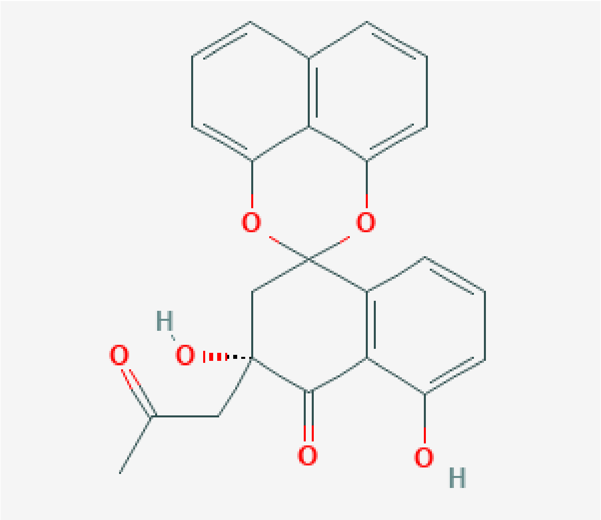

83. Guignardin B [64]

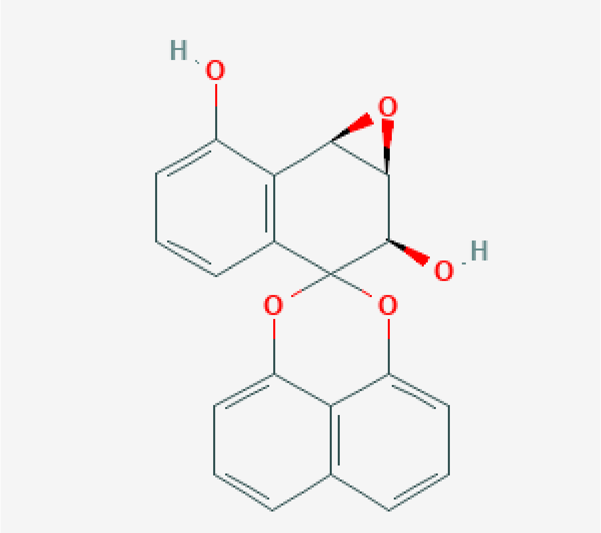

84. Guignardin C [65]

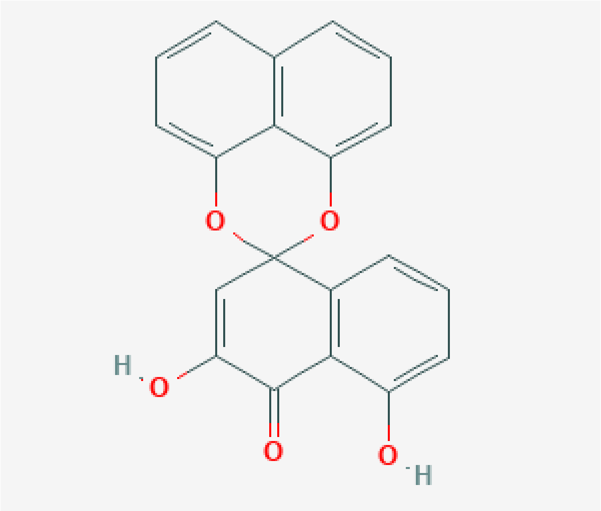

85. Guignardin D [66]

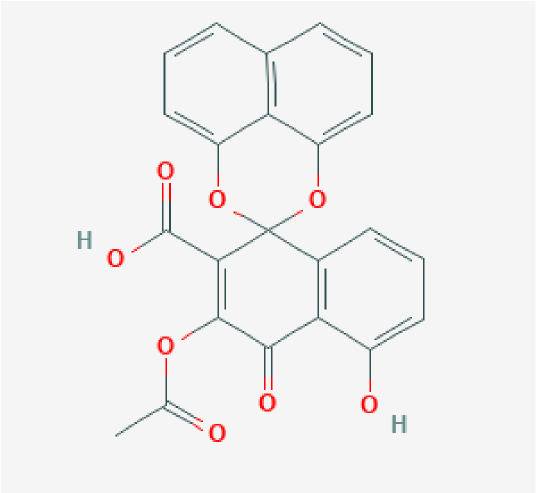

86. Guignardin E [67]

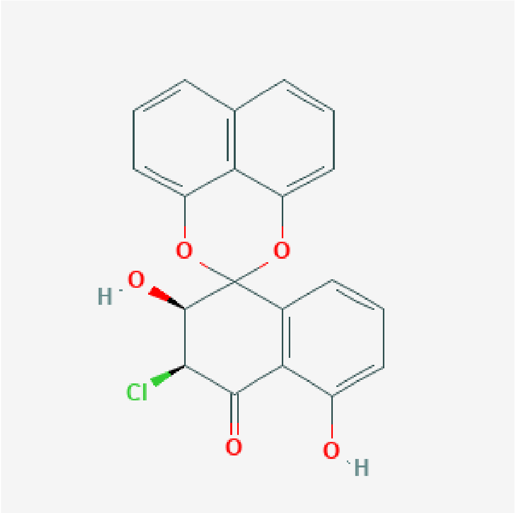

87. Guignardin F [68]

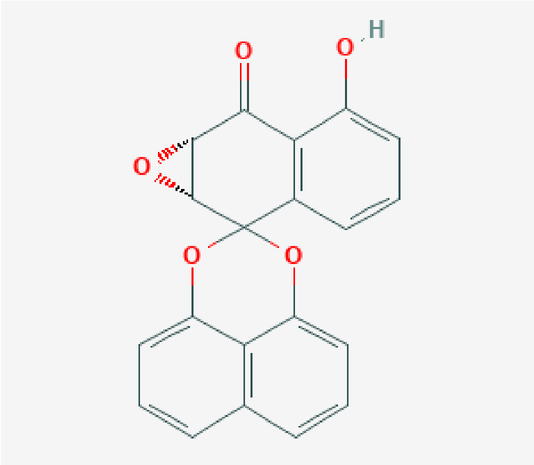

88. Palmarumycin C1 [69]

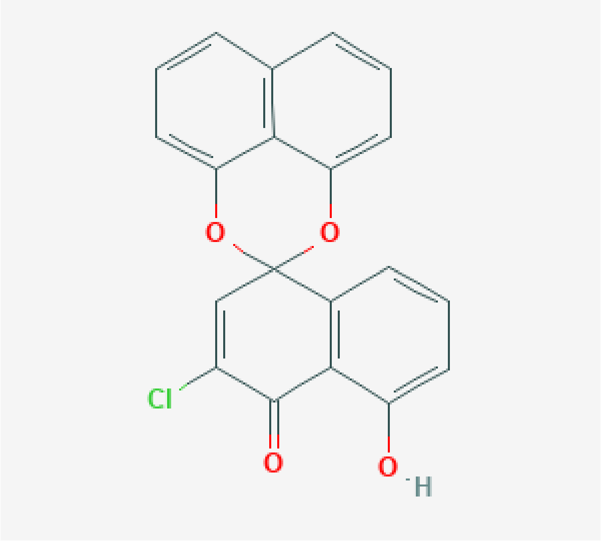

89. BG1

90. JC1[70]

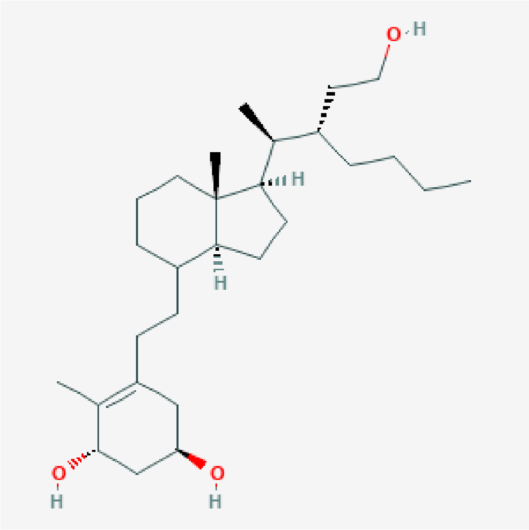

91. Lovastatin [71]

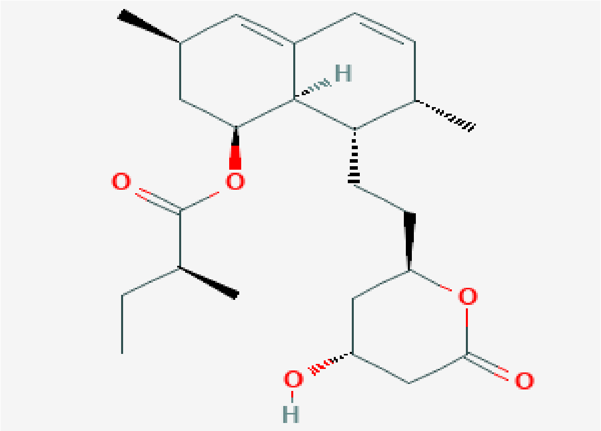

92. Protocatechuic acid [72]

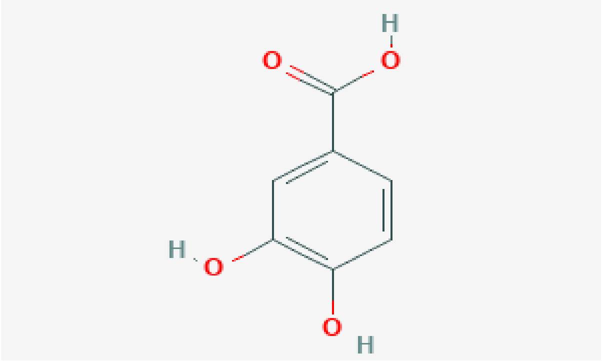

93. Acropyrone [73]

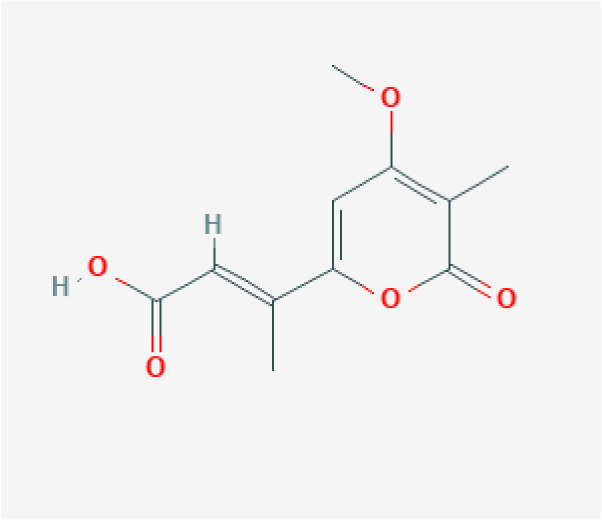

94. 1,4-Cyclohexadiene [74]

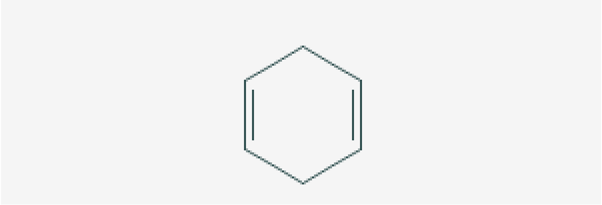

References

1. Zhou L, Zhao J, Xu L, et al. (2009) Antimicrobial compounds produced by plant endophytic fungi. *Fungic Chem Environ Impact Heal Eff* 91–119
2. National Center for Biotechnology Information (2021). PubChem Compound Summary for CID 18915, Ethyl 9-oxononanoate. Retrieved April 4, 2021 from https://pubchem.ncbi.nlm.nih.gov/compound/Ethyl-9-oxononanoate.
3. National Center for Biotechnology Information (2021). PubChem Compound Summary for CID 328789, 2(5H)-Furanone, 5-hydroxy-3,4-dimethyl-5-pentyl-. Retrieved April 4, 2021 from https://pubchem.ncbi.nlm.nih.gov/compound/2_5H_-Furanone_-5-hydroxy-3_4-dimethyl-5-pentyl.
4. National Center for Biotechnology Information (2021). PubChem Compound Summary for CID 5326970, Ergosta-5,7-dien-3beta-ol. Retrieved April 4, 2021 from https://pubchem.ncbi.nlm.nih.gov/compound/Ergosta-5_7-dien-3beta-ol.
5. National Center for Biotechnology Information (2021). PubChem Compound Summary for CID 68152, 3-Hydroxypropionic acid. Retrieved April 4, 2021 from https://pubchem.ncbi.nlm.nih.gov/compound/3-Hydroxypropionic-acid.
6. National Center for Biotechnology Information (2021). PubChem Compound Summary for CID 2758, Eucalyptol. Retrieved April 4, 2021 from https://pubchem.ncbi.nlm.nih.gov/compound/Eucalyptol.
7. National Center for Biotechnology Information (2021). PubChem Compound Summary for CID 36314, Paclitaxel. Retrieved April 4, 2021 from https://pubchem.ncbi.nlm.nih.gov/compound/taxol.
8. National Center for Biotechnology Information (2021). PubChem Compound Summary for CID 139583786, Phomopsichalasin. Retrieved April 4, 2021 from https://pubchem.ncbi.nlm.nih.gov/compound/Phomopsichalasin.
9. National Center for Biotechnology Information (2021). PubChem Compound Summary for CID 16078330, Cryptocandin. Retrieved April 4, 2021 from https://pubchem.ncbi.nlm.nih.gov/compound/Cryptocandin.
10. National Center for Biotechnology Information (2021). PubChem Compound Summary for CID 71362212. Retrieved April 4, 2021 from https://pubchem.ncbi.nlm.nih.gov/compound/71362212.
11. National Center for Biotechnology Information (2021). PubChem Compound Summary for CID 11066670. Retrieved April 4, 2021 from https://pubchem.ncbi.nlm.nih.gov/compound/11066670.
12. National Center for Biotechnology Information (2021). PubChem Compound Summary for CID 10935547. Retrieved April 4, 2021 from https://pubchem.ncbi.nlm.nih.gov/compound/10935547.
13. National Center for Biotechnology Information (2021). PubChem Compound Summary for CID 11012219, 7-Butyl-6,8-dihydroxy-3(R)-pentylisochroman-1-one. Retrieved April 4, 2021 from https://pubchem.ncbi.nlm.nih.gov/compound/7-Butyl-6_8-dihydroxy-3_R_-pentylisochroman-1-one.
14. National Center for Biotechnology Information (2021). PubChem Compound Summary for CID 101360525, Asperfumoid. Retrieved April 4, 2021 from https://pubchem.ncbi.nlm.nih.gov/compound/Asperfumoid.
15. National Center for Biotechnology Information (2021). PubChem Substance Record for SID 174522689, ASPERFUMIN, Source: ChEMBL. Retrieved April 4, 2021 from https://pubchem.ncbi.nlm.nih.gov/substance/174522689.

16. National Center for Biotechnology Information (2021). PubChem Compound Summary for CID 23872041, Monomethylsulochrin. Retrieved April 4, 2021 from https://pubchem.ncbi.nlm.nih.gov/compound/Monomethylsulochrin.
17. National Center for Biotechnology Information (2021). PubChem Compound Summary for CID 57339223, Fumigaclavine C. Retrieved April 4, 2021 from https://pubchem.ncbi.nlm.nih.gov/compound/Fumigaclavine-C.
18. National Center for Biotechnology Information (2021). PubChem Compound Summary for CID 403923, Fumitremorgin C. Retrieved April 4, 2021 from https://pubchem.ncbi.nlm.nih.gov/compound/Fumitremorgin-C.
19. National Center for Biotechnology Information (2021). PubChem Compound Summary for CID 10639, Physcion. Retrieved April 4, 2021 from https://pubchem.ncbi.nlm.nih.gov/compound/Physcion.
20. National Center for Biotechnology Information (2021). PubChem Compound Summary for CID 3002143, Helvolic acid. Retrieved April 4, 2021 from https://pubchem.ncbi.nlm.nih.gov/compound/Helvolic-acid.
21. An X, Feng BM, Chen G, et al (2016) Isolation and identification of two new compounds from marine-derived fungus Acremonium fusidioides RZ01. *Chin J Nat Med* 14:934–938.
22. National Center for Biotechnology Information (2021). PubChem Compound Summary for CID 444679, Ergosterol. Retrieved April 4, 2021 from https://pubchem.ncbi.nlm.nih.gov/compound/Ergosterol.
23. National Center for Biotechnology Information (2021). PubChem Compound Summary for CID 5287620, Brefeldin A. Retrieved April 4, 2021 from https://pubchem.ncbi.nlm.nih.gov/compound/Brefeldin-A.
24. National Center for Biotechnology Information (2021). PubChem Compound Summary for CID 54751220. Retrieved April 4, 2021 from https://pubchem.ncbi.nlm.nih.gov/compound/54751220.
25. National Center for Biotechnology Information (2021). PubChem Compound Summary for CID 159926, Macrosporin. Retrieved April 4, 2021 from https://pubchem.ncbi.nlm.nih.gov/compound/Macrosporin.
26. National Center for Biotechnology Information (2021). PubChem Compound Summary for CID 15071544, Citreoisocoumarin. Retrieved April 4, 2021 from https://pubchem.ncbi.nlm.nih.gov/compound/Citreoisocoumarin.
27. National Center for Biotechnology Information (2021). PubChem Compound Summary for CID 159926, Macrosporin. Retrieved April 4, 2021 from https://pubchem.ncbi.nlm.nih.gov/compound/Macrosporin.
28. National Center for Biotechnology Information (2021). PubChem Compound Summary for CID 24882465. Retrieved April 4, 2021 from https://pubchem.ncbi.nlm.nih.gov/compound/24882465.
29. National Center for Biotechnology Information (2021). PubChem Compound Summary for CID 24882470, Ampelanol. Retrieved April 4, 2021 from https://pubchem.ncbi.nlm.nih.gov/compound/Ampelanol.
30. National Center for Biotechnology Information (2021). PubChem Compound Summary for CID 89644, Altersolanol A. Retrieved April 4, 2021 from https://pubchem.ncbi.nlm.nih.gov/compound/Altersolanol-A.
31. National Center for Biotechnology Information (2021). PubChem Compound Summary for CID 195315, Alterporriol E. Retrieved April 4, 2021 from https://pubchem.ncbi.nlm.nih.gov/compound/Alterporriol-E.

32. National Center for Biotechnology Information (2021). PubChem Compound Summary for CID 10913856, Altersolanol J. Retrieved April 4, 2021 from https://pubchem.ncbi.nlm.nih.gov/compound/Altersolanol-J.
33. National Center for Biotechnology Information (2021). PubChem Compound Summary for CID 36314, Paclitaxel. Retrieved April 4, 2021 from https://pubchem.ncbi.nlm.nih.gov/compound/taxol.
34. National Center for Biotechnology Information (2021). PubChem Compound Summary for CID 5646, Usnic acid. Retrieved April 4, 2021 from https://pubchem.ncbi.nlm.nih.gov/compound/Usnic-acid.
35. National Center for Biotechnology Information (2021). PubChem Compound Summary for CID 131379, Cercosporamide. Retrieved April 4, 2021 from https://pubchem.ncbi.nlm.nih.gov/compound/Cercosporamide.
36. National Center for Biotechnology Information (2021). PubChem Substance Record for SID 274654506, Phomodione, Source: Japan Chemical Substance Dictionary (Nikkaji). Retrieved April 4, 2021 from https://pubchem.ncbi.nlm.nih.gov/substance/274654506.
37. National Center for Biotechnology Information (2021). PubChem Compound Summary for CID 6438581, Phomopsin A. Retrieved April 4, 2021 from https://pubchem.ncbi.nlm.nih.gov/compound/Phomopsin-A.
38. National Center for Biotechnology Information (2021). PubChem Compound Summary for CID 131752487, Phomopsin B. Retrieved April 4, 2021 from https://pubchem.ncbi.nlm.nih.gov/compound/Phomopsin-B.
39. National Center for Biotechnology Information (2021). PubChem Compound Summary for CID 24866623, Phomopsin C. Retrieved April 4, 2021 from https://pubchem.ncbi.nlm.nih.gov/compound/Phomopsin-C.
40. National Center for Biotechnology Information (2021). PubChem Compound Summary for CID 10687292, Cytosporone B. Retrieved April 4, 2021 from https://pubchem.ncbi.nlm.nih.gov/compound/Cytosporone-B.
41. National Center for Biotechnology Information (2021). PubChem Compound Summary for CID 10778975, Cytosporone C. Retrieved April 4, 2021 from https://pubchem.ncbi.nlm.nih.gov/compound/Cytosporone-C.
42. National Center for Biotechnology Information (2021). PubChem Compound Summary for CID 3663, Hypericin. Retrieved April 4, 2021 from https://pubchem.ncbi.nlm.nih.gov/compound/Hypericin.
43. National Center for Biotechnology Information (2021). PubChem Compound Summary for CID 345501, Deoxypodophyllotoxin. Retrieved April 4, 2021 from https://pubchem.ncbi.nlm.nih.gov/compound/Anthricin.
44. National Center for Biotechnology Information (2021). PubChem Compound Summary for CID 38358410, Xiamycin. Retrieved April 4, 2021 from https://pubchem.ncbi.nlm.nih.gov/compound/Xiamycin.
45. National Center for Biotechnology Information (2021). PubChem Compound Summary for CID 50898452, Xiamycin methyl ester. Retrieved April 4, 2021 from https://pubchem.ncbi.nlm.nih.gov/compound/Xiamycin-methyl-ester.
46. National Center for Biotechnology Information (2021). PubChem Compound Summary for CID 56840153, Benquinol. Retrieved April 4, 2021 from https://pubchem.ncbi.nlm.nih.gov/compound/Benquinol.

47. National Center for Biotechnology Information (2021). PubChem Compound Summary for CID 56924800, Benquoine. Retrieved April 4, 2021 from https://pubchem.ncbi.nlm.nih.gov/compound/Benquoine.
48. National Center for Biotechnology Information (2021). PubChem Compound Summary for CID 23275141, Terpene-3197-12. Retrieved April 4, 2021 from https://pubchem.ncbi.nlm.nih.gov/compound/Terpene-3197-12..
49. National Center for Biotechnology Information (2021). PubChem Compound Summary for CID 545574, 8-Octadecanone. Retrieved April 4, 2021 from https://pubchem.ncbi.nlm.nih.gov/compound/8-Octadecanone.
50. National Center for Biotechnology Information (2021). PubChem Compound Summary for CID 14260, 1-Tetradecene. Retrieved April 4, 2021 from https://pubchem.ncbi.nlm.nih.gov/compound/1-Tetradecene.
51. National Center for Biotechnology Information (2021). PubChem Compound Summary for CID 13162, 8-Pentadecanone. Retrieved April 4, 2021 from https://pubchem.ncbi.nlm.nih.gov/compound/8-Pentadecanone.
52. National Center for Biotechnology Information (2021). PubChem Compound Summary for CID 15712, Octylcyclohexane. Retrieved April 4, 2021 from https://pubchem.ncbi.nlm.nih.gov/compound/Octylcyclohexane.
53. National Center for Biotechnology Information (2021). PubChem Compound Summary for CID 10441, 10-Nonadecanone. Retrieved April 4, 2021 from https://pubchem.ncbi.nlm.nih.gov/compound/10-Nonadecanone.
54. National Center for Biotechnology Information (2021). PubChem Compound Summary for CID 53363661. Retrieved April 4, 2021 from https://pubchem.ncbi.nlm.nih.gov/compound/4-hydroxy-5_6-dimethoxy-2_3-dihydroisoindol-1-one.
55. National Center for Biotechnology Information (2021). PubChem Compound Summary for CID 53363662, Emerimidine B. Retrieved April 4, 2021 from https://pubchem.ncbi.nlm.nih.gov/compound/Emerimidine-B.
56. Zhang G, Sun S, Zhu T, et al (2011) Antiviral isoindolone derivatives from an endophytic fungus Emericella sp. associated with Aegiceras corniculatum. *Phytochemistry* 72: 1436–1442.
57. National Center for Biotechnology Information (2021). PubChem Compound Summary for CID 45359507, Aspernidine A. Retrieved April 4, 2021 from https://pubchem.ncbi.nlm.nih.gov/compound/Aspernidine-A.
58. National Center for Biotechnology Information (2021). PubChem Compound Summary for CID 46867924, Aspernidine B. Retrieved April 4, 2021 from https://pubchem.ncbi.nlm.nih.gov/compound/Aspernidine-B.
59. National Center for Biotechnology Information (2021). PubChem Compound Summary for CID 38353601, Austin. Retrieved April 4, 2021 from https://pubchem.ncbi.nlm.nih.gov/compound/Austin.
60. National Center for Biotechnology Information (2021). PubChem Compound Summary for CID 56955927, Austinol. Retrieved April 4, 2021 from https://pubchem.ncbi.nlm.nih.gov/compound/Austinol.
61. National Center for Biotechnology Information (2021). PubChem Compound Summary for CID 122201239, Dehydroaustin. Retrieved April 4, 2021 from https://pubchem.ncbi.nlm.nih.gov/compound/Dehydroaustin.

62. National Center for Biotechnology Information (2021). PubChem Compound Summary for CID 101660521, Acetoxydehydroaustin. Retrieved April 4, 2021 from https://pubchem.ncbi.nlm.nih.gov/compound/Acetoxydehydroaustin.
63. National Center for Biotechnology Information (2021). PubChem Compound Summary for CID 101904185, Guignardin A. Retrieved April 4, 2021 from https://pubchem.ncbi.nlm.nih.gov/compound/Guignardin-A.
64. National Center for Biotechnology Information (2021). PubChem Compound Summary for CID 101904186, Guignardin B. Retrieved April 4, 2021 from https://pubchem.ncbi.nlm.nih.gov/compound/Guignardin-B.
65. National Center for Biotechnology Information (2021). PubChem Compound Summary for CID 101904187, Guignardin C. Retrieved April 4, 2021 from https://pubchem.ncbi.nlm.nih.gov/compound/Guignardin-C.
66. National Center for Biotechnology Information (2021). PubChem Compound Summary for CID 101904188, Guignardin D. Retrieved April 4, 2021 from https://pubchem.ncbi.nlm.nih.gov/compound/Guignardin-D.
67. National Center for Biotechnology Information (2021). PubChem Compound Summary for CID 101904189, Guignardin E. Retrieved April 4, 2021 from https://pubchem.ncbi.nlm.nih.gov/compound/Guignardin-E.
68. National Center for Biotechnology Information (2021). PubChem Compound Summary for CID 9905621, Deoxypreussomerin A. Retrieved April 4, 2021 from https://pubchem.ncbi.nlm.nih.gov/compound/Deoxypreussomerin-A.
69. National Center for Biotechnology Information (2021). PubChem Compound Summary for CID 101672314, Palmarumycin C1. Retrieved April 4, 2021 from https://pubchem.ncbi.nlm.nih.gov/compound/Palmarumycin-C1.
70. National Center for Biotechnology Information (2021). PubChem Compound Summary for CID 25150850. Retrieved April 4, 2021 from https://pubchem.ncbi.nlm.nih.gov/compound/25150850.
71. National Center for Biotechnology Information (2021). PubChem Compound Summary for CID 53232, Lovastatin. Retrieved April 4, 2021 from https://pubchem.ncbi.nlm.nih.gov/compound/Lovastatin.
72. National Center for Biotechnology Information (2021). PubChem Compound Summary for CID 72, 3,4-Dihydroxybenzoic acid. Retrieved April 4, 2021 from https://pubchem.ncbi.nlm.nih.gov/compound/3_4-Dihydroxybenzoic-acid.
73. National Center for Biotechnology Information (2021). PubChem Compound Summary for CID 102367304, Acropyrone. Retrieved April 4, 2021 from https://pubchem.ncbi.nlm.nih.gov/compound/Acropyrone.
74. National Center for Biotechnology Information (2021). PubChem Compound Summary for CID 12343, 1,4-Cyclohexadiene. Retrieved April 4, 2021 from https://pubchem.ncbi.nlm.nih.gov/compound/1_4-Cyclohexadiene.

AIMS Press

© 2021 the Author(s), licensee AIMS Press. This is an open access article distributed under the terms of the Creative Commons Attribution License (http://creativecommons.org/licenses/by/4.0)
